# Diagnostic and clinical utility of whole genome sequencing in a cohort of undiagnosed Chinese families with rare diseases

**DOI:** 10.1038/s41598-019-55832-1

**Published:** 2019-12-18

**Authors:** Hong-Yan Liu, Liyuan Zhou, Meng-Yue Zheng, Jia Huang, Shu Wan, Aiying Zhu, Mingjie Zhang, Anliang Dong, Ling Hou, Jia Li, Haiming Xu, Bingjian Lu, Weiguo Lu, Pengyuan Liu, Yan Lu

**Affiliations:** 1 Department of Medical Genetics, Henan Provincial People’s Hospital, People’s Hospital of Zhengzhou University, People’s Hospital of Henan University, Zhengzhou, Henan 450003 China; 2 Department of Respiratory Medicine, Sir Run Run Shaw Hospital and Institute of Translational Medicine, School of Medicine, Zhejiang University, Hangzhou, Zhejiang, 310016 China; 3 Women’s Reproductive Health Key Laboratory of Zhejiang Province, Department of Gynecologic Oncology, Women’s Hospital and Institute of Translational Medicine, School of Medicine, Zhejiang University, Hangzhou, Zhejiang 310006 China; 4 Department of Neurosurgery, The First Affiliated Hospital, School of Medicine, Zhejiang University, Hangzhou, Zhejiang 310003 China; 5 Department of Dermatology, People’s Hospital of Lushi County, Lushi, Henan 472200 China; 6 Institute of Bioinformatics, Zhejiang University, Hangzhou, Zhejiang 310058 China; 7 Center of Systems Molecular Medicine, Department of Physiology, Medical College of Wisconsin, Milwaukee, WI 53226 USA

**Keywords:** Computational biology and bioinformatics, Clinical genetics, Genetic testing

## Abstract

Rare diseases are usually chronically debilitating or even life-threatening with diagnostic and therapeutic challenges in current clinical practice. It has been estimated that 80% of rare diseases are genetic in origin, and thus genome sequencing-based diagnosis offers a promising alternative for rare-disease management. In this study, 79 individuals from 16 independent families were performed for whole-genome sequencing (WGS) in an effort to identify the causative mutations for 16 distinct rare diseases that are largely clinically intractable. Comprehensive analysis of variations, including simple nucleotide variants (SNVs), copy-number variations (CNVs), and structural variations (SVs), was implemented using the WGS data. A flexible analysis pipeline that allowed a certain degree of misclassification of disease status was developed to facilitate the identification of causative variants. As a result, disease-causing variants were identified in 10 of the 16 investigated diseases, yielding a diagnostic rate of 62.5%. Additionally, new potentially pathogenic variants were discovered for two disorders, including IGF2/INS-IGF2 in mitochondrial disease and FBN3 in Klippel–Trenaunay–Weber syndrome. Our WGS analysis not only detected a CNV associated with 3p deletion syndrome but also captured a simple sequence repeat (SSR) variation associated with Machado–Joseph disease. To our knowledge, this is the first time the clinical WGS analysis of short-read sequences has been used successfully to identify a causative SSR variation that perfectly segregates with a repeat expansion disorder. After the WGS analysis, we confirmed the initial diagnosis for three of 10 established disorders and modified or corrected the initial diagnosis for the remaining seven disorders. In summary, clinical WGS is a powerful tool for the diagnosis of rare diseases, and its diagnostic clarity at molecular levels offers important benefits for the participating families.

## Introduction

Rare disease, defined as a disease that affects no more than 200,000 (about 1 in 1,500) people in the United States or less than 1 in 2,000 in Europe^1^, collectively affects millions of people worldwide (>300 million according to Global Gene Corp). The number of rare diseases is estimated at roughly 7,000 and most of them are usually chronically debilitating or even life threatening, posing heavy burdens on the affected families and society^2^. Notwithstanding the severity, rare diseases have not drawn much attention for many years due to their individual rarity, and they have been regarded as health orphans. Fortunately, public awareness of rare diseases has increased over the last three decades, owing to the efforts of worldwide patient support groups. To date, over half of recorded rare diseases have been determined from their molecular etiology using traditional linkage mapping or candidate gene analysis, although this process may take a long period and incur great expenditure with respect to both labor and resources^3^. On the other hand, many rare diseases present a challenge for these conventional gene discovery methods because of the scarcity of affected samples for powerful analysis.

The advances in next-generation sequencing (NGS) technology^4,5^ largely surmount these issues and have ushered in a new era for disease-gene discovery. Estimates suggest that ~80% of rare diseases are genetic, and thus they are considered suitable for study using NGS technology. In 2009, Ng *et al*. first evaluated the performance of this new technology in disease-gene discovery using a rare disorder called Freeman–Sheldon syndrome with a known causative gene *MYH3* as the proof-of-concept^6^. This study showed that genomic sequencing analysis could rapidly find the causative gene using a small number of samples (four unrelated, affected individuals in this case) without any linkage or disease mechanism information. Inspired by this successful validation study, Ng *et al*. applied this approach to other rare genetic disorders with unknown genetic cause in the following year and successfully discovered the pathogenic genes for Miller syndrome^7^ and Kabuki syndrome^8^. Meanwhile, other research groups also achieved exciting results using a similar strategy for gene discovery in rare diseases such as Schinzel–Giedion syndrome^9^ and Fowler syndrome^10^ etc. Of note, some pathogenic variants identified by these studies were found to be *de novo*, which are almost impossible to identify by conventional linkage analysis. In a recent study^11^, the NGS technology was used for deciphering the prevalence and architecture of *de novo* mutations (DNMs) in developmental disorders (DDs). It was found that DNMs account for approximately half of the genetic architecture of severe DDs.

While NGS has been massively and successfully applied to discovery of rare-disease-causing genes, an NGS-based rare disease clinic seemingly remains to be developed. In this study, 79 individuals from 16 independent families were recruited for whole-genome sequencing (WGS) in an effort to identify causative mutations for 16 distinct rare human diseases that are largely clinically intractable. Our study highlighted the utility of clinical WGS and proposed solutions for more accurate diagnosis in the context of rare diseases.

## Results

### Patient samples

**Figure 1:**
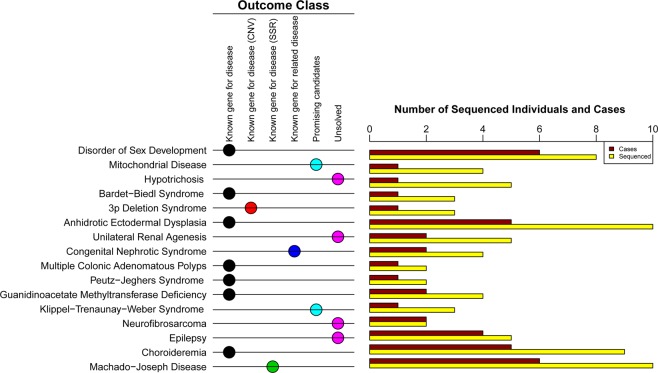
Overview of the study and outcomes.

Thirty-two affected families were initially collected via the genetic services of Henan Provincial People’s Hospital and Women’s Hospital of Zhejiang University. These families presented distinct rare diseases with a broad range of symptoms, including neurogenic, sterile, developmental and renal disorders, etc. Routine clinic workups or treatments were carried out for these cases but the effect was not evident with most of them even failing to be diagnosed. Molecular genetic tests, such as target gene sequencing and array comparative genomic hybridization, were also performed for some cases subsequently in the clinic while most cases remained unexplained in the current clinical setting. In consideration of the low success rate with the long testing periods of the previous genetic tools, WGS analysis, which can characterize all types of genetic variations in the genome in a short time, was adopted to study these unsolved cases. The families that were eligible to participate in the clinical WGS analysis met the following three criteria: (1) Their disease causes were not resolved after routine clinical workups and/or molecular genetic tests; (2) DNA samples in multiple family members are also available; and (3) Patients and their family members were consented into the WGS analysis. As a result, 16 unsolved families consisting of 79 individuals qualified for entry into the clinical WGS analysis in the study (Table S1 and Fig. S1). Altogether, at least one case from each family was chosen for WGS analysis. Healthy controls, which were the closest relative of those affected (such as parents of cases, as long as their DNA samples are available and qualified for library preparation), were also chosen for WGS analysis (Fig. 1).

### Whole genome sequencing analysis

We conducted WGS analysis of blood DNA for 79 subjects using the HiSeq X10 for sequencing with 150 bp paired-end reads. The output short reads were aligned to the human reference genome (GRCh37/hg19) using the Burrows–Wheeler Aligner (BWA)^12^. We obtained approximately 28.5 (±9.4 SD) fold mean sequence coverage after removing low-quality sequences and PCR duplications (Table S2). Using the Genome Analysis Toolkit (GATK)^13^, variant calling was performed for each sample individually using the HaplotypeCaller in gVCF mode, and then joint variant calling was conducted on these gVCFs to increase genotyping accuracy (Fig. S2). The variants produced from the joint-call cohort were used for subsequent variant filtration. On average, approximately 4.7 million (ranging from 4,432,484 to 4,936,343) raw single nucleotide variants (SNV) and small insertion and deletion (INDEL) changes were identified for each personal genome in comparison to the human reference genome.

### Variant filtering and annotation

After the joint variant calling, we analyzed putative causative variants for each rare disease. We assumed that causal variants associated with rare diseases are rare and highly unlikely presented in other pedigrees in our studied cohort (i.e., “rare disease rare variant” hypothesis). Therefore, for a specific affected family, unaffected members of that family and all of the sequenced subjects from the other families were treated as healthy controls. First, according to the manifestation mode of a disease within the family, SNVs and INDELs were filtered based on one or more of the four patterns of disease inheritance, dominant, recessive caused by a homozygous variant or by two compound heterozygous variants, X-linked, and *de novo* mutations. Second, the resulting lists of SNVs and INDELs were then annotated using ANNOVAR^14^. Variants that are present in the 1000 Genomes (1000G) Project with high frequency were removed when any one of the following conditions meet: (1) at least one population with frequency >=0.05; (2) at least two populations with frequency >=0.03; (3) at least three populations with frequency >=0.025; an (4) at least four populations with frequency >= 0.02. Finaly, we used multiple software tools, including SIFT, PolyPhen, LRT, MutationTaster, MutationAssessor, FATHMM, MetaSVM, and MetaLR, to predict whether the non-silent variants affected protein function^15^. Deleterious variants were defined as those whose number of tools supporting “deleterious”, “probably damaging”, “functional” or “disease-causing” overwhelmed those supporting the others (Table S3). After the above multistep filtration, for most cases only 0–5 variants were retained for each inheritance mode.

**Figure 2:**
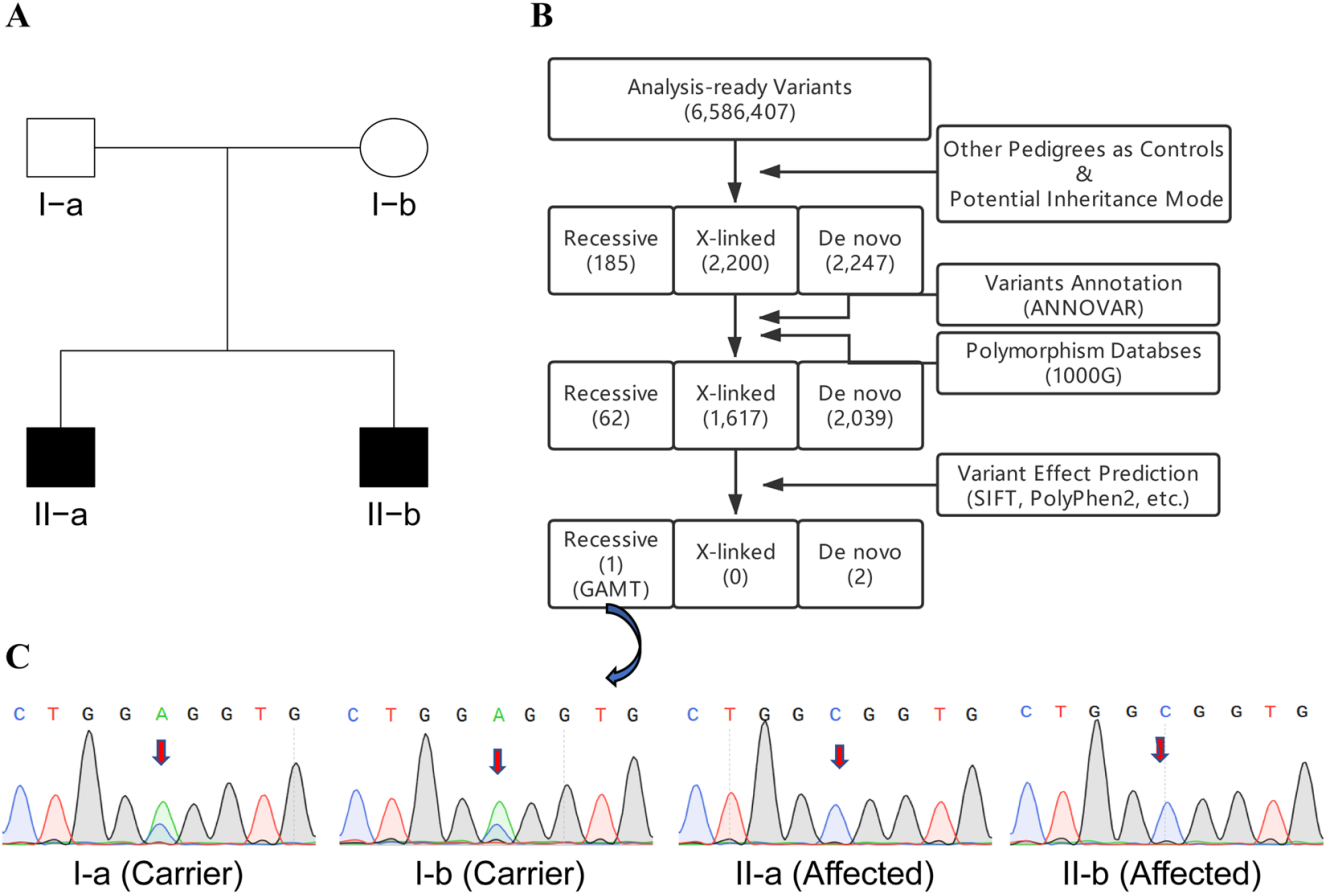
A schema of the variant filtering and selection for identifying pathogenic mutations in GAMT predisposing to guanidinoacetate methyltransferase deficiency. (**A**) Pedigree of Fam24 with guanidinoacetate methyltransferase deficiency disease. (**B**) Variant filtering and selection procedure. (**C**) Sanger sequencing validation of a missense mutation in GAMT.

Taking the disease presented in the tetrad Fam24 with two affected sons (Fig. 2A) as an example: The potential modes of inheritance in Fam24 are recessive, X-linked and *de novo* according to its pedigree information. The disorder is rare and its associated risk variant is likely rare and the risk variant of Fam24 is highly unlikely presented in other pedigrees in the studied cohort. Therefore, all subjects sequenced in other families in the same cohort were also treated as controls. Variants were filtered out, when either they are presented in any of control samples, or they are not fitted to any of potential modes of inheritance (i.e., recessive, X-linked and de novo). As a result, there were 185, 2,200, and 2,247 variants retained for recessive, X-linked, and *de novo* modes, respectively (Fig. 2B). Then these variants were annotated and those presenting in the polymorphism database were removed, which led to a further reduction of candidate variants (62 for recessive, 1,617 for X-linked, and 2,039 for *de novo*, separately) (Fig. 2B). Finally, the variant effect was predicted using multiple tools and only those predicted deleterious variants were kept. After this step, only 1 variant residing in the GAMT gene was retained for recessive mode and 1 variant residing in each DSPP and ZNF628 genes were retained for *de novo* mode. Finally, the variant located in the GAMT gene was identified as the pathogenic variant according to the integrated information, and Sanger sequencing validation was carried out for further confirmation (Fig. 2C).

### Overview of the findings

We identified variants with high confidence for 10 of 16 investigated diseases, including nine known causative genes for the corresponding disorders and one known gene for a related disorder of the target disease (Table 1 and Fig. 1). These variants included a variety of mutation types: three missense variants, two nonsense variants, two splicing variants, one copy-number variant (CNV), one simple sequence repeat (SSR) variant, and one compound heterozygous mutation with a nonsense and a frameshift deletion variant. Notably, the CNV and SSR variant are detectable by WGS rather than whole-exome sequencing (WES). The inheritance modes of these diseases were also various, comprising three autosomal dominant (AD), two autosomal recessive (AR), two X-linked recessive (XLR), one CNV *de novo*, one compound heterozygous, and one SSR autosomal dominant (Table 1). In addition, we discovered promising candidates for two disorders, and failed to figure out the remaining four disorders (Table 2). All reporting variants were further confirmed using Sanger sequencing. In addition, the pLI relating score^16^, which indicates the degree that a gene is intolerant to a Loss of Function (LoF) mutation, was assessed for the reported 13 genes as an additional confirmation of the deleteriousness of identified variants (Table S4). The closer score is to one, the more LoF intolerant the gene appears to be.

**Table 1:** Summary of 10 disorders with established high reliable causative variants.

Family	Initial Diagnosis	Final Diagnosis	Gene	Chr.	Position (−)	Position (+)	Ref./Mut.	Mutation Type	Inheritance Mode
5-1	46,XY sex reversal	Disorder of Sex Development	NR5A1	9	127265357	127265357	C/T	splicing	AD
10-1	Bardet-Biedl Syndrome	Bardet-Biedl Syndrome	TTC8(BBS8)	14	89327564	89327564	A/G	splicing	AR
10-2	No diagnosis	3p Deletion Syndrome	CNV	3	0	10349999	—	CNV(loss)	De novo
13	No diagnosis (exclued the Anhidrotic Ectodermal Dysplasia)	Anhidrotic Ectodermal Dysplasia	EDA	X	69176954	69176954	A/C	missense	XLR
21	Membranoproliferative Glomerulonephritis	Congenital Nephrotic Syndrome	UPK3A	22	45683310	45683311	CT/-	frameshift_deletion	CHT
				22	45684998	45684998	G/A	nonsense	
22-1	Peutz-Jeghers syndrome	Peutz-Jeghers syndrome	STK11	19	1219406	1219406	C/A	missense	AD
22-2	Adenomatous Polyposis Coli	Adenomatous Polyposis Coli	APC	5	112128143	112128143	C/T	nonsense	AD
24	Mental Retadation with Seizures	Guanidinoacetate methyltransferase deficiency	GAMT	19	1399922	1399922	T/G	missense	AR
28	Retinitis Pigmentosa	Choroideremia	CHM	X	85213886	85213886	G/A	nonsense	XLR
32	No diagnosis	Machado-Joseph Disease	ATXN3	14	92537362	92537378	—	SSR	AD

Note: Ref. = reference allele; Mut. = mutation allele; AD = autosomal dominant; AR = autosomal recessive; CHT = compound heterozygous; XLR = X-linked recessive.

**Table 2:** Summary of two disorders with promising candidate variants.

Famlily	Initial Diagnosis	Candidate Gene	Chr.	Position (−)	Position (+)	Ref./Mut.	Mutation Type	Inheritance Mode
7	Mitochondrial Disease	BCKDHA	19	41928938	41928938	C/T	missense	de novo
		IGF2, INS-IGF2	11	2170355	2170355	C/T	splicing	imprinted (paternally expressed)
25	Klippel-Trenaunay-Weber Syndrome	FBN3	19	8188820	8188820	C/T	missense	AR

Note: Ref. = reference allele; Mut. = mutation allele; AR = autosomal recessive.

### Causative SNV variants

To test the robustness of our in-house analysis pipeline for detecting causative SNV variants, we included a case of adenomatous polyposis coli which is known to be caused by inactivation of the APC gene. As expected, our analysis confirmed a nonsense variant in the APC gene in this patient (Fam22-2). Using our analysis pipeline, we identified causative SNV variants for four disorders on the first pass (Table 1). These included a homozygous splicing variant in TTC8/BBS8 for Bardet–Biedl syndrome (Fam10-1), a heterozygous missense variant in STK11 for Peutz–Jeghers syndrome (Fam22-1), a homozygous missense variant in GAMT for guanidinoacetate methyltransferase deficiency disease (Fam24), and two compound heterozygous variants in UPK3A for congenital nephrotic syndrome (Fam21). Of note, the case in Fam24 was initially diagnosed as mental retardation and was subsequently corrected as guanidinoacetate methyltransferase deficiency disease after the identification of a recessive mutation in GAMT which was further confirmed by clinical re-examination. In Fam21 with congenital nephrotic syndrome, both affected children carried two heterozygous mutations in UPK3A.

### Misclassification of disease status

Due to the rarity and heterogeneity of phenotypes, misclassification of case and control is not uncommon during clinical diagnosis of rare diseases. This could lead to the failure of identification of potential causative variants. To address this issue, the analysis pipeline was designed to allow a certain degree of misclassification of disease status. That is, we assumed that a certain number of cases were classified incorrectly into controls and/or a certain number of controls were classified incorrectly into cases in the analyzed family. Using this misclassification tolerant pipeline, we reanalyzed the unresolved families on a case-by-case basis. The results from each of re-classification analyses were subsequently subject to manual literature curation and retracing investigation of diagnostic ambiguity. Such strategy led to identification of causative SNV variants for 3 additional rare diseases, including disorder of sex development (Fam5-1), anhidrotic ectodermal dysplasia (Fam13), and choroideremia (Fam28, initially diagnosed as retinitis pigmentosa) (Table 1).

**Figure 3:**
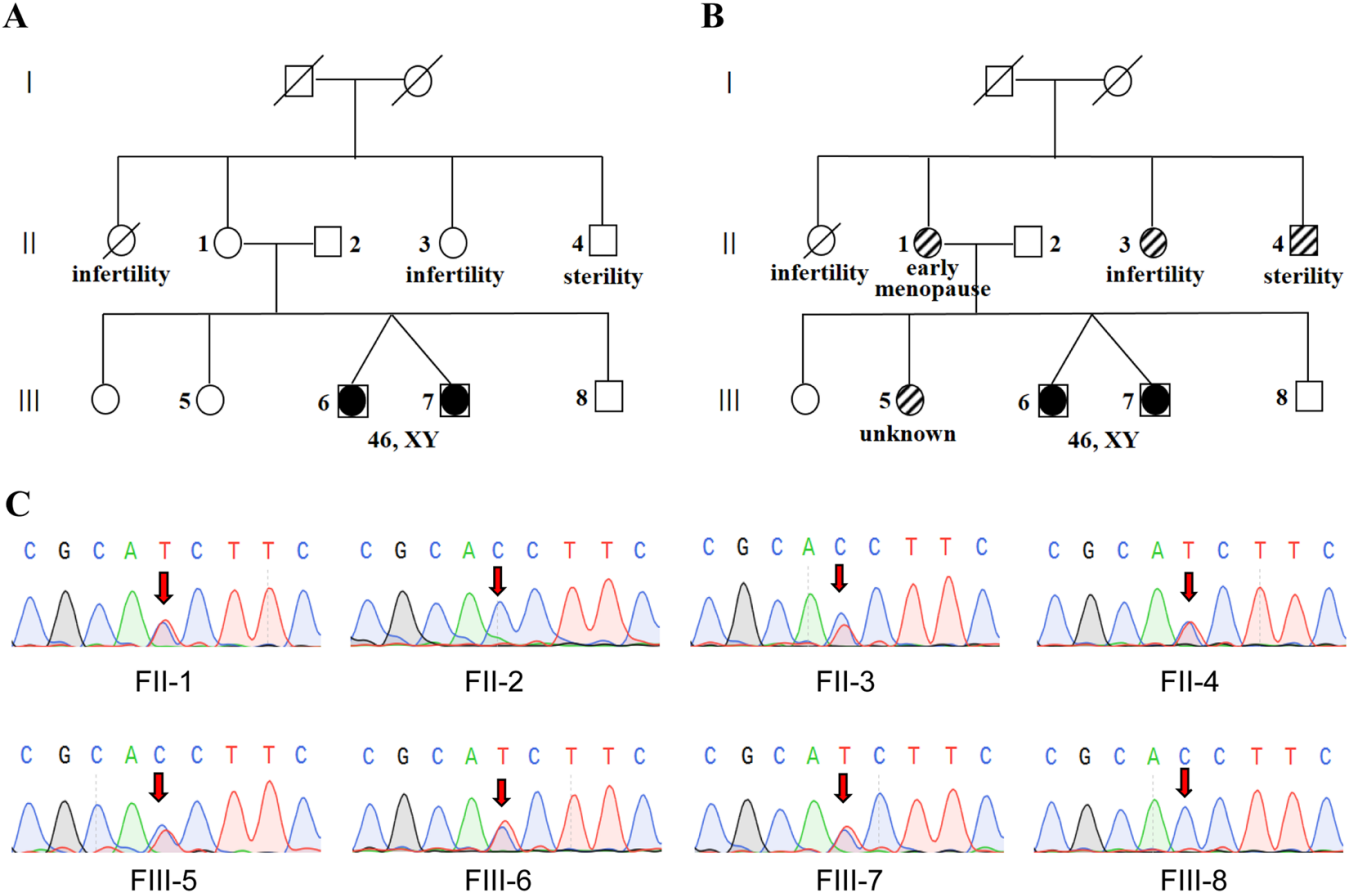
Improving disease-gene discovery by correcting diagnostic misclassification of patient samples. (**A**) Pedigree and phenotypic heterogeneity of Fam5-1 with disorder of sex development. (**B**) Corrected classification resulted in identifying causative variants. (**C**) Sanger sequencing validation of a splicing mutation in NR5A1.

In Fam5-1, there was a large inherent phenotypic variability in the disorder of sex development. Available clinical phenotypes included two female probands (twins) that were 46, XY sex reversal; their two aunts were 46, XX infertility (one alive and one dead without available DNA); and their only uncle was 46, XY sterility. A total of 8 subjects in Fam5-1 were chosen for the WGS analysis (Fig. 3A). Our initial analysis set the twins as cases and the other 6 individuals as controls, but no promising candidates whose variant status was consistent with any of the disease inheritance modes were found. However, our re-analysis allowing a certain amount of misclassification discovered a strong SNV within a critical splice site of NR5A1 (NM_004959:exon4:c.244 + 1 G > A). Six sequenced subjects carried this splicing mutation, including two probands (twins, sex reversal), their aunt (infertility) and uncle (sterility), their mother (seemingly normal) and sister (seemingly normal), while the other two sequences subjects, that is, their father (normal) and brother (normal), did not carry that mutation (Fig. 3B,C). In a previous study^17^, heterozygous NR5A1 gene mutations had been shown affecting the carriers with extreme within-family phenotypic divergence from 46, XX early menopause to 46, XY sex reversal or hypospadias. As we would expect, our further retracing investigation revealed their seemingly normal mother had early menopause at 37 years old; whereas, their sister who carried the NR5A1 mutant allele was perhaps too young (at 30 years old) to show any obvious symptoms.

For the other two disorders, the misclassification came from the artificial bias. Using our pipeline, we found strong candidates for both disorders where only one individual was not amenable to a genotype-phenotype pattern. They were a missense variant in EDA for anhidrotic ectodermal dysplasia (Fam13) and a nonsense variant in CHM for choroideremia (Fam28) (Table 1 and Figs. S1 and S3). Subsequent retracing investigation revealed that the unmatched individual in Fam13 that was initially wrongly recorded as unaffected by clinicians but carried the mutant allele based on the WGS analysis was actually affected with the disorder of anhidrotic ectodermal dysplasia. The unmatched individual in Fam28 that did not carry the mutant allele based on the WGS analysis but was self-reported as affected with choroideremia actually had normal myopia. It seemed that she had mistakenly reported her symptoms, which had most likely occurred because of the psychological effect arising from her multiple family members suffering from the disorder. These cases highlight the power of our robust pipeline, as well as the importance of comprehensive and detailed phenotyping.

### Causative structural variants

Using the SNV filtering pipeline, we identified relatively confirmative causative SNVs for eight rare disorders. For the remaining eight unexplained disorders, the gapless WGS data enabled us to carry out copy-number variant and structural variant analysis.

**Figure 4:**
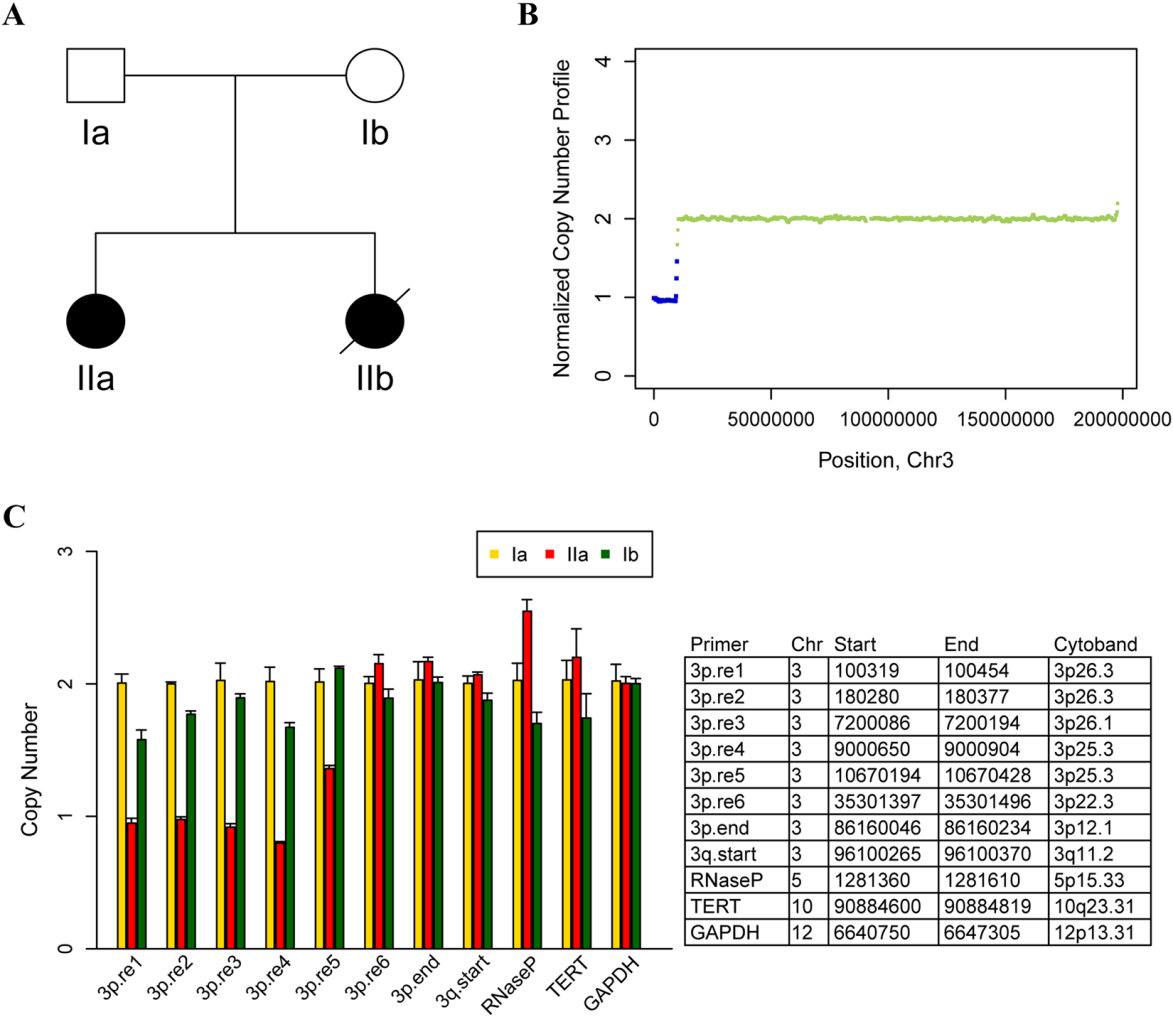
Identification of a CNV associated with severe abnormality of multiple organs and systems. (**A**) Pedigree of Fam10-2 with 3p deletion syndrome. (**B**) CNVs detected on chromosome 3 of the affected. (**C**) Quantitative PCR validation of a 3p deletion.

In Fam10-2 (Fig. 4A), where the clinical features of the proband were characterized as polydactyly, severe mental and developmental retardation, congenital spina bifida, gastric volvulus, and nerve reflex insensitivity, the primary diagnosis of the referring clinician was descriptive information related to abnormality in multiple organs and systems. Through CNV analysis, we detected a *de novo* ~10 Mb loss at the pter of chromosome 3 (chr3:0-10349999) in the patient (Fig. 4B) and subsequently corrected the diagnosis as a 3p deletion syndrome. The subsequent quantitative real time PCR experiment confirmed the existence of this *de novo* loss (Fig. 4C).

**Figure 5:**
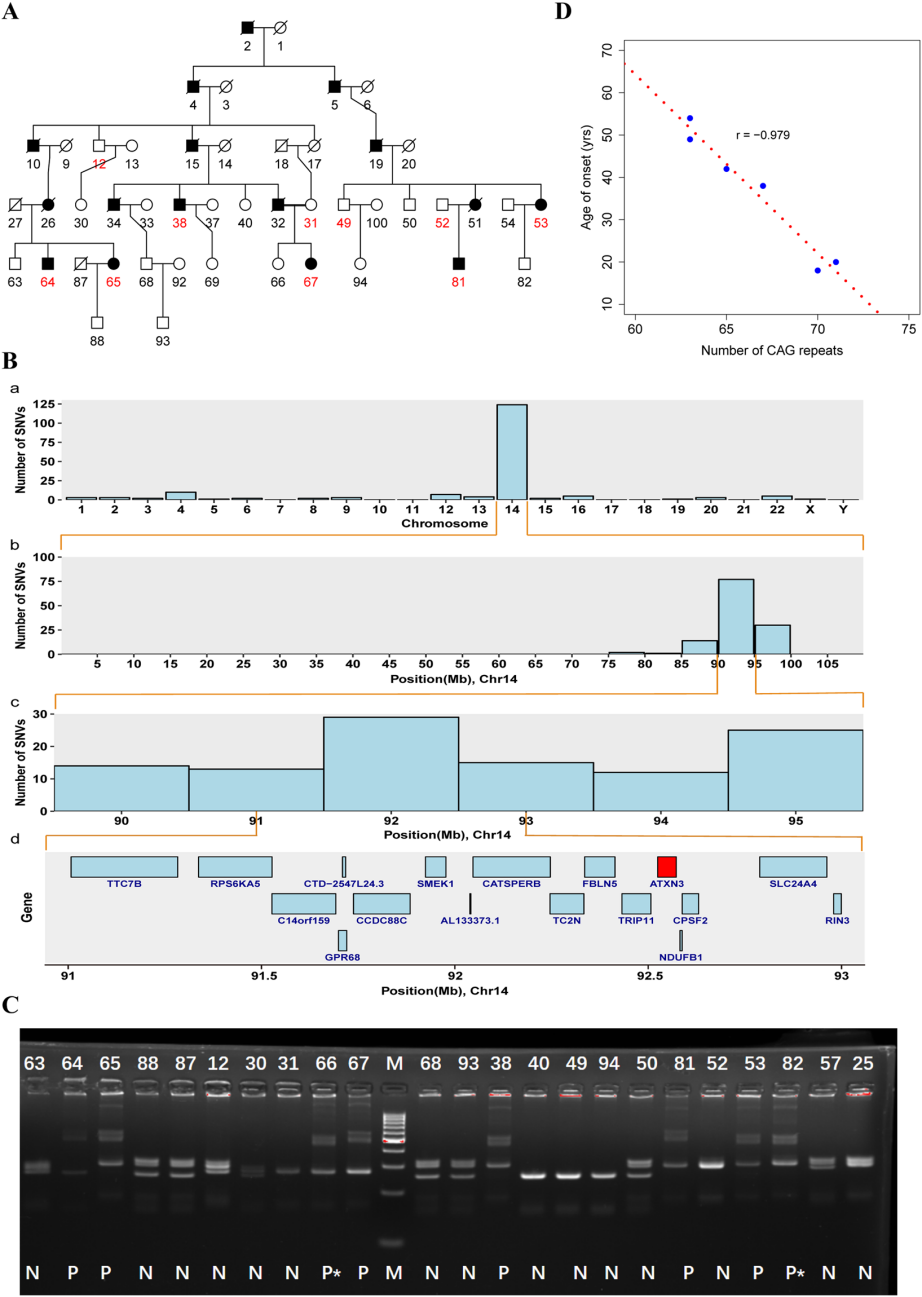
Identification of an SSR associated with Machado–Joseph disease. (**A**) Pedigree of Fam32 with Machado–Joseph disease. (**B**) (a) The distribution of number of detected variants across the genome; (b) The distribution of number of detected variants on chromosome 14; (c) Enhanced view of chr14 89.5-95.5 Mb region; (d) Genes located within chr14 91–93 Mb region. (**C**) PCR validation of SSR in ATXN3; P = Patient, N = Normal, P* = Potential patient who is young and does not exhibit obvious symptoms but carries abnormal CAG repeats. (**D**) The number of CAG repeats is negatively correlated with the age of onset.

Patients in Fam32 were diagnosed as having an odd and difficult disease that had haunted many generations of the family. This disease was characterized by progressive symptoms including muscle weakness, muscle convulsion, dysarthria, and difficulty swallowing. The onset of age varied, ranging from 18 to 54 years old, and 10 patients were dead and six patients were alive (Fig. 5A). Ten individuals including six living patients and four healthy relatives in this multigenerational family were recruited for the WGS analysis. After the SNV filtering pipeline, we did not find any candidates that satisfied the criteria for variant reporting. However, we noticed an interesting phenomenon where 142 variants were retained after stage 3 filtering (i.e., 1000G), of which 116 variants were located on chromosome 14 (Fig. 5B-a). Furthermore, all of these variants were co-segregated with the disease status. On chromosome 14, we found 110 of 116 variants were enriched in ~79–95 Mb region (Fig. 5B-b) and in this region the central point of the enriched peak was at 92 Mb (Fig. 5B-c). These results suggested that these co-segregating variants may be in linkage disequilibrium with a nearby unknown casual variant. We thus exhaustively examined genes within the 92 ± 1 Mb (91–93 Mb) region and found a strong candidate gene ATXN3 (Fig. 5B-d). Through PCR analysis, followed by Sanger sequencing and high performance capillary electrophoresis (HPCE), we confirmed that the expansion of an unstable CAG tract in exon 10 of the ATXN3 was a causal mutation for the disease called Machado–Joseph Disease (MJD), a type of spinocerebellar ataxia (type 3, SCA3). The confirmatory experiment showed that patients had two different allelic genes, one was normal, but the other one had more than 66 CAG repeats (Fig. 5C). The (CAG)n tail of the normal allele was GGG while the disease allele showed polymorphisms. Furthermore, we found that the number of CAG repeats was negatively correlated with the age of onset (Figs. 5D and S4).

### New potentially pathogenic candidates

We identified several candidate variants, which were retained after the variant filtration and prioritization, and the Sanger sequencing validation, in two cases, which included mitochondrial disease in Fam7, and Klippel–Trenaunay–Weber syndrome in Fam25. However, these candidate variants were not located in the related known disease-causing genes and are potentially new pathogenic variants for these rare disorders (Table 2).

For Fam7 with the initial diagnosis of mitochondrial disease (Table S1), a *de novo* missense variant in BCKDHA (BCKDHA:NM_000709:exon8:c.C1031T:p.A344V, BCKDHA:NM_001164783:exon8:c.C1028T:p.A343V) was retained after the multistep filtration. The protein encoded by BCKDHA is the alpha subunit of the decarboxylase (E1α) component of the branched-chain alpha-keto acid dehydrogenase complex, which is an inner mitochondrial enzyme complex that catalyzes the second major step in the catabolism of the branched-chain amino acids leucine, isoleucine, and valine. It was reported that homozygous or compound heterozygous mutations in BCKDHA would result in maple syrup urine disease (MSUD)^18–20^ which is supposed to be an autosomal recessive disease while the variant in the present case is a heterozygous mutation in BCKDHA, and also, the clinical symptoms of this case were not consistent with MSUD. These results suggest that the missense variant in BCKDHA is not a strong candidate predisposing to Fam7.

Interestingly, another heterozygous variant affecting splicing of both IGF2 and INS-IGF2 was identified in Fam7 although both the patient and her normal father carried the mutation. This variant was located within a maternally imprinted (paternally expressed) region. The mutated allele of the normal father was further confirmed as inherited from his mother, which is not supposed to be expressed. The protein encoded by IGF2 is a potent mitogenic peptide hormone that is a member of the insulin family of polypeptide growth factors, which play key roles in mammalian growth, development, and metabolism^21,22^. INS-IGF2 is a read-through gene whose 5′ region overlaps the INS gene and the 3′ region overlaps the IGF2 gene and its function in humans is still under study^23^. INS-IGF2 has only recently been reported to be expressed primarily in human pancreatic beta cells and its protein is recognized as a novel autoantigen in Type 1 diabetes^24^. In contrast, the dysregulation of IGF2 has been widely reported to be associated with numerous human diseases notably Beckwith–Wiedemann overgrowth syndrome^25^, Silver–Russell syndrome (mainly characterized by prenatal and postnatal growth retardation)^26^, and various cancers^27^. The main symptoms of the present case were dystonia with curved fingers and legs, mental retardation, language disorder, but with seemingly normal body growth. However, these reported diseases are due mainly to epigenetic mutations of IGF2 while in our case it was a splicing mutation that likely disrupted the function of IGF2/INS-IGF2, which merits further investigation. Additionally, a *de novo* CNV loss (Chr7:100290000–100379999) including three genes (POP7, EPO, ZAN) was observed in the patient.

For Fam-25 with an initial diagnosis of Klippel–Trenaunay–Weber syndrome (KTWS), only recessively inherited mutations located in the FBN3 gene were detected among all types of variation analysis. FBN3 encodes a member of the fibrillin protein family, which are the extracellular matrix molecules that assemble into microfibrils in many connective tissues. Variants in FBN3 were reported to be associated with polycystic ovary syndrome^28,29^. No studies have established direct evidence linking FBN3 to KTWS. This implies that the uncharacterized function of FBN3 potentially contributes to KTWS.

## Discussion

The present study set out to identify causative variants for 16 rare diseases which are clinically intractable using WGS. Ultimately, we established high-confidence disease-causing variants for 10 disorders with a diagnostic yield of 62.5% (Table 1 and Fig. 1), which is comparable to that reported in recent clinical WGS/WES studies^30–35^ (Table S5). However, this high rate was not achieved on the first pass where the primary routine filtering only established the pathogenicity of five disorders. Retrospective analysis revealed that the correct classification of case and normal group is a significant contributing factor to the success rate. Apart from the artificial bias, which could be controlled to a great degree by careful examination, ambiguous classification of a disease is attributed to its inherent phenotypic variability. Such phenotypic divergence is likely to erode the success rate of previous studies. In this study, we developed a flexible analysis pipeline that allowed a certain degree of misclassification in order to overcome this challenge thus achieving promising outcomes. Furthermore, the WGS approach that enabled a comprehensive analysis of various variation types is another significant factor increasing the success rate. Using the WGS data, we not only detected a CNV associated with 3p deletion syndrome (Fig. 4) but also captured an SSR variation associated with Machado–Joseph disease (Fig. 5). To the best of our knowledge, this is the first time clinical WGS analysis using short-read sequencing has been used successfully to identify a causative SSR variation that perfectly segregates with a repeat expansion disorder. This result also suggests that the abnormal distribution of WGS variants on the genome stemming from co-segregation with the disease phenotype could be an indicator of SSR variation.

Because of the individual rarity, there is limited knowledge and experience with rare-disease management in clinical practices, and in many cases, even making a correct diagnosis is difficult. In the present study, before ordering the WGS analysis, referring physicians had carried out clinical diagnostic workups and made initial diagnoses for these disorders. However, these diagnoses were usually uncertain or categorical while most patients and their families were eager to receive a confirmed diagnosis. After clinical WGS analysis, we confirmed the initial diagnosis for three of 10 established disorders and modified or corrected the initial diagnosis for the remaining seven disorders (Table 1). This diagnostic clarity at molecular levels provided significant benefits for the participating families.

First, prenatal diagnosis could be adopted for all 10 resolved families for their healthy progeny. In some cases where clinicians had no diagnosis (in Machado–Joseph disease and 3p deletion symptom cases) or even excluded the correct diagnosis because of artificial bias (in the hypohidrotic ectodermal dysplasia case), our results cleared up the confusion for both patients and clinicians. Moreover, our clinical WGS analysis provides additional clinical utility for at least four participating families (Fam5-1, Fam28, Fam24 and Fam21). For Fam5-1, the revelation of a splicing variant in NR5A1 in 6 of 8 sequenced individuals (Fig. 3), where the initial diagnosis only identified 2 individuals with 46, XY sex reversal, not only resolved the confusion surrounding the varied symptoms (female infertility, male sterility, 46,XY sex reversal, etc.,) arising in this family but also incidentally unearthed two subtle patients with not so obvious symptoms of early menopause which in turn emphasized their need to pursue prenatal screening for healthy progeny although they seems normal. In a choroideremia-suffering family (Fam28), initially misdiagnosed as retinitis pigmentosa, a normal nearsighted girl was anxious about loss of vision in light of her older affected family members while our definitive molecular diagnosis eased her anxiety. Furthermore, the therapy could be achieved via the molecular diagnostic clarity in some cases. For the two children affected with guanidinoacetate methyltransferase deficiency (initially categorically diagnosed as mental retardation with seizures in Fam24), an inborn error of creatine biosynthesis characterized by creatine depletion and accumulation of guanidinoacetate (GAA) in the brain, they could supplement creatine in pharmacologic doses to restore the depleted creatine in the brain and the treatment effects could be further improved by dietary arginine restriction and ornithine supplementation (reduced seizures)^36^. In addition, some inappropriate treatments could be avoided resulting from the correct diagnosis, as shown by the family suffering from vesico-ureteral reflux (Fam21), where one of two affected children had died from drug complications. These results implied that the WGS-based causative-variant identification efficiently helped clinic diagnostic clarity and had significant benefits for both patients and their families.

We also acquired several novel promising candidates for two disorders, however the establishment of pathogenicity in these disorders still needs follow-up functional studies (Table 2). Unfortunately, we failed to find any significant variants responsible for the remaining four disorders. Most of these failures were attributed to small sample size and incomplete phenotyping of patients. Though the requirement for the total sample size for NGS study is much lower than that for conventional gene discovery methods, the “structure” of these samples (i.e., effective sample size) is still important to capture causative variants efficiently, especially when there is not very much solid knowledge about the gene-disease associations.

Lastly, it is interesting to estimate the cost-effectiveness of WGS compared with WES. In the present study, causal variants were identified in protein coding regions in 8 of 10 resolved families, which can be potentially resolved by WES. In China, the cost for WES sequencing and library preparation (~$330 per sample) is approximately 1/3 of WGS (~$1000 per sample). The cost ratio remains similar for the data analysis of WES and WGS. Therefore, in comparison to WES, the diagnostic rate of WGS is increased from 50% to 62.5%, but its cost increases three times. Thus the cost effectiveness of WGS should be evaluated for routine clinical diagnosis, although WGS is more powerful on detection of potential disease-causing mutations than WES even within protein coding regions^37^.

In summary, clinical WGS is a powerful tool for the diagnosis of rare diseases, and its diagnostic clarity at molecular levels offers important benefits for the participating families. The high diagnostic yield of clinical WGS for rare diseases was attributed to its capability of fully characterizing various variation types and the improved analysis strategy that allowed a certain degree of misclassification of disease status to facilitate the identification of causative variants.

## Methods and Materials

### Studied samples

Initially, 32 independent families were recruited from Zhejiang and Henan provinces in the study. These families presented distinct rare diseases with a broad range of symptoms. Routine clinical workups or treatments were carried out for these cases. Molecular genetic tests, such as target gene sequencing and array comparative genomic hybridization, were also performed for some cases subsequently in the clinic. However, more than half of the cases failed to be diagnosed or their symptoms could not be explained in the current medical setting. As a result, 16 unsolved families were qualified for entry into the clinical WGS analysis in the study (Fig. S1). To maximize the success rate while minimizing the sequencing cost, at least one case from each family was chosen for WGS analysis; healthy controls that were the closest relative of the affected were also chosen for WGS analysis. In total, 79 individuals from these independent families were chosen for WGS analysis. The study protocols were approved by the Institutional Review Boards of The First Affiliated Hospital and The Women’s Hospital of Zhejiang University (Hangzhou, China) and Henan Provincial People’s Hospital (Zhengzhou, China). All subjects provided informed consent to participate in the study. All experiments and methods were performed in accordance with the relevant guidelines and regulations.

### Sequencing library preparation

Genomic DNA was extracted from blood samples of patients using PureLink Genomic DNA Mini Kits (Thermo Fisher, USA). Paired-end libraries with insert sizes ranging from 350–450 bp were constructed with TruSeq Nano DNA Library Prep Kit from Illumina (Illumina, USA) according to the manufacturer’s instructions. The concentration and size distribution of the libraries were determined on an Agilent Bioanalyzer DNA 1000 chip. These libraries were sequenced on an Illumina HiSeq X Ten platform (Illumina, CA, USA) using the PE-150 module.

### Read mapping and alignment

We recently developed an in-house analysis pipeline for whole genome sequencing data (Fig. S2). Briefly, the adapter sequences were removed from the output short reads; sequences with low quality (base quality <13) at both ends of reads were trimmed, and the trimmed reads with less than 25 bp were further removed using FASTQC (https://www.bioinformatics.babraham.ac.uk/projects/fastqc/). The trimmed reads were aligned to a reference genome (GRCh37/hg19) using the BWA^12^. Each alignment was assigned a mapping quality score by the BWA, which is the Phred-scaled probability that the alignment is incorrect. The PCR duplicates were detected and removed by Picard (https://github.com/broadinstitute/picard).

### Variant calling, annotation and filtering

After alignment, joint SNV (SNPs and INDELs) variant calling of these samples was performed using the GATK best practices workflow^13^. Copy number variations (gain and loss) were detected using the Control-FREEC^38^. The Kolmogorov–Smirnov test was used for evaluating the significance of CNVs (q-values < 0.05). Structural variants (large deletion, insertion, inversion and intra- and inter-chromosomal translocation) were identified by the Clipping REveals STructure (CREST) algorithm with default parameters^39^. SVs were filtered with at least one soft-clipped read in both sides of the breaking points.

After variant calling, these variants were filtered based on one of four patterns of disease inheritance: dominant, recessive caused by a homozygous variant or by two compound heterozygous variants, X-linked, and *de novo* mutation. The resulting list of variants was annotated using ANNOVAR^14^, classifying into frameshift and non-frameshift INDELs, missense, nonsense and splicing mutations. Then, they were further filtered with dbSNP, the 1000 Genomes Project, and NHLBI-ESP Exome Consortium. Finally, we used multiple software tools, including SIFT, PolyPhen, LRT, MutationTaster, MutationAssessor, FATHMM, MetaSVM, and MetaLR, to predict whether the non-silent variants affect protein function^15^. Deleterious variants were defined as those whose number of tools supporting “deleterious”,“probably damaging”, “functional” or “disease-causing” overwhelmed those supporting the others.

All SNVs, CNVs and SSRs retained after the multistep filtration in the WGS analysis were chosen for experimental validation (Supplementary Materials).

### Ethics approval and consent to participate

The study protocols were approved by the Institutional Review Boards of The First Affiliated Hospital and The Women’s Hospital of Zhejiang University (Hangzhou, China) and Henan Provincial People’s Hospital (Zhengzhou, China). All subjects provided informed consent to participate in the study.

## Supplementary information

Supplementary Materials

## Data Availability

Many of these samples studied in our paper were consented for clinical investigation only. Some of these samples were collected for general research, and whole-genome sequencing data (fastq files) for these samples were deposited in the Genome Sequence Archive (GSA) under accession HRA000091 (http://bigd.big.ac.cn/gsa-human).
