# Supplementary material for: Diagnostic and clinical utility of whole genome sequencing in a cohort of undiagnosed Chinese families with rare diseases
Source: Sci Rep. 2019 Dec 18;9:19365. doi: 10.1038/s41598-019-55832-1 (PMC6920370; doi:10.1038/s41598-019-55832-1)
Supplement: Supplementary file 1 — Supplementary Materials [file 41598_2019_55832_MOESM1_ESM.pdf]

Diagnostic and clinical utility of whole genome sequencing in a cohort of undiagnosed Chinese families with rare diseases

Hong-Yan Liu, Liyuan Zhou, Meng-Yue Zheng, Jia Huang, Shu Wan, Aiying Zhu, Mingjie Zhang, Anliang Dong, Ling Hou, Jia Li, Haiming Xu, Bingjian Lu, Weiguo Lu, Pengyuan Liu, Yan Lu

Supplementary Materials

Supplementary Materials

Supplementary Materials and Methods

Table S1. A brief description of clinical features of probands in 16 families.

Table S2. Quality control metrics of 79 WGS samples.

Table S3. Summary of categorical criteria for nine variant effect prediction tools.

Table S4. The pLI relating scores for 13 analyzed genes.

Table S5. Summary of diagnostic rates for several recent WES/WGS studies.

Fig. S1. Pedigrees of 16 families with rare disorders. Squares represent males and circles represent females. Squares or circles filled with black represent the affected. Squares or circles with a slash represent the deceased. The individuals with red text are selected for sequencing.

Fig. S2. Bioinformatics pipeline of read alignment and variant calling. Briefly, the Genome Analysis Toolkit (GATK) was adopted for read alignment and variant calling. Variant calling was performed for each sample individually using the HaplotypeCaller in gVCF mode. Then, joint variant calling was conducted on these gVCFs to increase genotyping accuracy. The variants produced from the joint-call cohort were used for subsequent variant filtration.

Fig. S3. PCR and Sanger validation results. For a specific Sanger sequencing result, each of four bases is recorded at top with its own representative color as shown in the figure. Below, the single peak indicates the homozygous state of the above base and bimodal peak indicates the heterozygous state of bases interpreted by the colors of peaks. Red arrow indicates the position of target variant in Sanger validation sequence.

Fig. S4. HPCE analysis of SSR in ATXN3. The horizontal coordinate represents the length of PCR products, and the vertical coordinate represents the concentration of PCR products. The yellow peak is the DNA marker that marked corresponding location information, which assists to normalize the length of input PCR products. Blue peaks are our input PCR products from normal people and patients. Single peak represents homozygous genotype while bimodal peaks represent heterozygous genotype of people. The PCR products of normal people are within 26-40 CAG repeats, while patients have one longer unstable PCR products within more than 60 CAG repeats.

Supplementary Materials and Methods

Experimental Validation

All SNVs, CNVs and SSRs retained after the multistep filtration in the WGS analysis were chosen for experimental validation. We performed Sanger sequencing using the ABI 3730 sequencer to validate SNVs, real-time fluorescent quantitative PCR using the ViiA^{TM} 7 Real-time PCR System to validate CNVs, and high performance capillary electrophoresis (HPCE) using the Agilent 7100 System to validate the SSR.

The standard PCR was conducted in a 20μl reaction volume that contained 10μl 2X PCR Solution Premix (Takara Code No. RR003Q), 20 pM of each primer, and about 20 ng camel genome DNA. The PCR reaction conditions were set as: 94°C for the first 5 min, followed by 35 cycles of 94°C denaturation for 30s, 60°C annealing for 30s, and 72°C extension for 30–60s. The PCR were accompanied by negative controls containing the reaction solutions without DNA. The primers for PCR reaction were used for sequencing.

The real-time quantitative PCR was conducted in a 20μl reaction volume that contained 10μl 2x TB Green Premix Ex Taq, 0.4ul ROX Reference Dye II, 0.4ul 10μm F/R primers and 20ng camel genome DNA. The qPCR reaction conditions were set as: 95°C pre-degenerated for the first 30s, followed by 40 cycles of 95°C denaturation for 5s, and 60°C annealing and extension for 34s. In the qPCR validated procedure, we used universal internal control GAPDH, TERT and GAPDH. In addition, we also validated these regions of the adjacent unmutated areas, respectively, both in the long arm and short arm in the same chromosome.

The standard PCR in HPCE was conducted in a 10μl reaction volume that contained 5μl 2X PCR Solution Premix (Takara Code No. RR003Q), 10 pM of each primer, and about 10 ng camel genome DNA, adding a hold step of 72°C for 10 min in the end to increase fluorescence stability. The F primer was synthesized with FAM in the 5' end by Applied Biosystems^{TM}

.

Table S1. A brief description of clinical features of probands in 16 families.

Family	Age	proband	Sex	Race	Age of Onset	Affected System	Presenting Phenotype	Initial Diagnosis	Previous Genetic Testing	Final Diagnosis	Age at Return of WGS Results	Impact on Medical Management
5-1	22	III-d	F	East Asian	15	Endocrine, Reproductive	Primary amenorrhea, infertility, infantile uterus (B-ultrasonography, at the age of 15)	46,XY sex reversal	Karyotype	Disorder of Sex Development	24	Assisted reproduction, Prenatal diagnosis
7	7	II-a	F	East Asian	Neonatal period	Neuromuscular	Dystonia with curved finger and leg, mental retardation, language disorder,seemingly normal body growth	Mitochondrial Disease	Metabolic disease Panel; Mitochondrial Nuclear gene Panel; Array-CGH	No diagnosis	/	/
8	24	II-a	M	East Asian	7	Skin	Losing hair gradually at the age of about 7; regrowing new hair at the age of about 12 but then falling off again; now all the hair, eyebrows, and pubic hair are out of light	Hypotrichosis	/	No diagnosis	/	/
10-1	16	II-a	M	East Asian	At birth	Skin, Skeletal, Eye, Reproductive	Polydactyly, obesity, vitiligo, retinitis pigmentosa, cryptorchidism	Bardet-Biedl Syndrome	/	Bardet-Biedl Syndrome	17	Prenatal diagnosis
10-2	4	II-a	F	East Asian	At birth	Central Nervous, Skeletal, Endocrine	Polydactyly, congenital spina bifida, gastric volvulus, congenital hypothyroidism, and nerve reflex insensitivity, severe mental and developmental retardation	No diagnosis	/	3p deletion syndrome	6	Prenatal diagnosis
13	30	III-f	F	East Asian	In childhood	Skin	Hypodontia, anhidrosis, rigid spine, hypotrichosis,	No diagnosis	/	Ectodermal Dysplasia	31	Prenatal diagnosis
20	28	II-a	M	East Asian	At birth	Urinary	Unilateral renal	No diagnosis	/	No diagnosis	/	/
21	2	II-a	M	East Asian	2	Urinary	Proteinuria 3+(2.0-4.0g/L), palpebral oedema	Membranoproliferative glomerulonephritis	/	Vesicoureteral reflux	4	Prenatal diagnosis
22-1	24	II-f	F	East Asian	5	Colonic, Skin	Multiple colonic adenomatous polyps, melanocytic macules on the lips, recurrentcolicky abdominal pain	Peutz-Jeghers Syndrome	/	Peutz-Jeghers Syndrome	25	Prenatal diagnosis,Tumor early screen
22-2	6	III-b	M	East Asian	4	Colonic	Multiple colonic adenomatous polyps	Adenomatous Polyposis Coli	/	Adenomatous Polyposis Coli	8	Prenatal diagnosis,Tumor early screen
24	15	II-a	M	East Asian	neonatal period	Central Nervous	Intellectual disability, epileptic seizure, aphasia, cannot take self-care activities	Intellectual disability with Seizures	Array-CGH	Guanidinoacetate methyltransferase deficiency	16	Prenatal diagnosis, Increase Creatine intake
25	7	II-a	F	East Asian	5	Skin, Skeletal	Cutaneous hemangiomata, capillary hemangioma, 2nd macrodactyly of foot	Klippel-Trenaunay-Weber Syndrome	/	No diagnosis	/	/
26	25	II-a	F	East Asian	neonatal period	Skin	Multiple neurofibromatosis, multiple hemangioma, chromatosis	Neurofibrosarcoma	/	No diagnosis	/	/
27	7	IV-c	M	East Asian	5 month	Central Nervous	Epilepsy, autism, Intellectual disability , language disorder	Idiopathic Epilepsy	/	No diagnosis	/	Antiepileptic treatment
28	65	III-g	M	East Asian	In childhood	Eye	Peripheral vision loss, blurred vision, childhood night blindness, gradual loss of vision	Retinitis pigmentosa	/	Choroideremia	67	Prenatal diagnosis
32	34	V-i	F	East Asian	18	Cerebellar Atrophy	Muscle convulsion, progressive symptoms including muscle weakness, speech fuzzy, and difficult swallowing	No diagnosis	/	Machado-Joseph Disease	36	Prenatal diagnosis

Table S2. Quality control metrics of 79 WGS samples.

Sample ID	Yield (Gb)	# Reads (M)	% of >= Q30 Bases (PF)	Mean Quality Score (PF)	% of mapping rate	Coverage* (median+/-SD)
24-199	96.5	643.4	92.3	36.2	94.2	25.6 ± 8.9
24-200	94.7	631.2	91.2	35.4	93.7	24.8 ± 8.8
24-201	107.8	718.5	92.8	36.2	94.3	28.6 ± 9.7
24-202	95.0	633.5	90.9	35.3	93.7	24.8 ± 8.6
5-1-9	95.9	639.6	93.5	36.2	94.3	25.6 ± 8.9
5-1-10	119.4	795.9	94.5	36.9	94.5	31.4 ± 10.1
5-1-13	98.7	657.9	94.4	36.7	94.4	26.3 ± 9
5-1-16	111.1	740.6	94.6	36.9	94.1	29.4 ± 10.2
5-1-17	113.8	758.9	94.2	36.8	94.7	30.6 ± 9.5
5-1-18	111.2	741.0	93.5	36.5	94.5	29.7 ± 9.8
5-1-20	104.6	697.2	93.5	36.4	95.0	28.4 ± 9.1
5-1-22	99.0	660.2	93.3	36.4	94.8	26.8 ± 8.7
7-45	91.9	612.7	92.6	36.1	94.3	24.7 ± 8.7
7-46	123.9	825.9	93.5	36.5	94.6	33 ± 10.9
7-47	104.3	695.4	92.2	36.0	94.9	28 ± 9.2
7-48	106.5	710.3	93.3	36.4	94.9	28.8 ± 9.4
8-49	96.4	642.3	94.9	37.2	94.5	26.6 ± 9.1
8-50	112.1	747.5	96.0	37.6	95.3	31.4 ± 9.9
8-51	106.0	706.5	95.5	37.5	94.6	29.4 ± 9.8
8-52	104.7	698.0	95.1	37.0	94.4	29 ± 9.9
10-1-54	117.5	783.4	95.7	37.5	95.3	32.8 ± 10.1
10-1-55	103.3	688.8	94.6	36.9	94.3	28.4 ± 9.6
10-1-56	112.8	752.2	96.0	37.6	94.9	31.4 ± 10.3
10-2-57	108.6	724.3	95.8	37.6	95.1	30.6 ± 9.7
10-2-58	104.4	696.1	95.5	37.5	95.2	29.3 ± 9.3
10-2-59	116.4	775.9	93.7	36.9	94.6	31.1 ± 10.2
13-60	125.4	835.9	93.9	37.0	95.0	33.6 ± 10.4
13-65	110.5	736.4	94.2	37.0	94.8	29.9 ± 9.7
22-2-191	91.3	608.7	91.4	35.8	93.5	24 ± 8.6
25-204	110.8	738.5	93.3	36.7	94.5	29.5 ± 9.9
25-205	92.0	613.6	92.1	36.3	94.6	24.6 ± 8.2
26-206	97.3	649.0	93.3	36.7	95.2	26.5 ± 8.7
27-211	98.9	659.1	93.4	36.8	95.2	27 ± 8.9
27-212	101.6	677.6	93.3	36.7	94.3	27.1 ± 9.2
27-213	108.8	725.5	93.5	36.9	94.9	28.8 ± 9.2
27-214	105.7	704.3	93.1	36.7	94.8	28 ± 9.5
28-220	102.4	682.6	93.2	36.7	94.9	27.3 ± 8.9
28-221	127.7	851.5	93.4	36.8	94.6	33.1 ± 10.8
28-223	105.4	702.8	91.8	36.3	94.8	27.6 ± 8.9
28-224	111.1	740.7	93.7	36.9	95.0	29.4 ± 9.3
28-227	90.0	599.6	93.1	36.7	94.6	23.8 ± 8
28-239	110.5	736.6	93.9	37.0	94.8	29 ± 9.7
28-240	115.8	771.9	93.2	36.8	94.8	30.6 ± 10.1
13-68	116.0	773.2	90.0	35.4	94.2	30 ± 10.2
13-69	96.6	644.2	89.6	35.3	94.3	25.2 ± 8.9
13-71	99.3	662.0	89.7	35.3	94.6	26.1 ± 8.6
13-73	96.4	642.4	89.8	35.3	94.4	25.2 ± 8.8

Table S2. Quality control metrics of 79 WGS samples (continued).

20-169	95.0	633.1	89.7	35.1	94.2	24.7 ± 8.4
20-170	98.4	656.0	91.0	35.7	94.6	25.8 ± 8.9
20-171	104.3	695.3	89.5	35.0	93.6	26.6 ± 9.3
20-172	112.0	746.6	93.2	36.7	94.5	29.4 ± 9.4
21-173	95.5	636.6	92.6	36.5	94.7	25.4 ± 8.8
21-174	103.7	691.5	92.9	36.6	94.9	27.5 ± 9.3
21-175	121.2	807.7	93.1	36.7	94.7	32 ± 10.5
22-1-179	133.4	889.1	93.4	36.8	94.8	34.8 ± 10.7
22-1-182	107.3	715.3	93.4	36.8	94.8	28.4 ± 9.2
13-66	106.3	708.4	90.3	35.0	95.0	24.1 ± 8.9
13-62	132.0	879.89	98.0	39.2	95.2	31.8 ± 9.8
13-63	109.6	730.65	98.0	39.1	95.8	27 ± 8.2
13-64	137.8	918.87	98.1	39.2	95.6	33.6 ± 10.3
20-245	122.6	817.50	91.1	35.4	95.0	23 ± 7.9
21-176	135.5	903.37	98.0	39.2	96.0	33 ± 9.4
22-2-192	94.0	626.33	98.0	39.2	95.5	23 ± 7.4
25-203	136.4	909.46	98.1	39.2	95.6	32.9 ± 9.5
26-207	134.3	895.24	98.0	39.2	95.7	31.9 ± 9.4
27-217	89.6	597.55	97.8	39.1	95.0	21.9 ± 7.4
28-219	102.5	683.28	91.2	35.7	93.7	22.6 ± 8.1
28-244	91.3	631.36	90.8	35.3	94.3	18.1 ± 6.6
8-53	145.6	970.97	98.2	39.2	95.3	35.1 ± 10.6
32-1	119.8	798.91	92.3	36.1	93.9	30.1 ± 10.3
32-2	121.0	806.88	92.8	36.2	94.2	30.5 ± 10
32-9	119.1	793.89	91.9	36.0	93.8	29.6 ± 10.2
32-10	119.9	799.04	91.8	36.0	94.1	29.7 ± 10.1
32-11	115.0	766.96	92.4	36.1	94.7	29.1 ± 9.6
32-13	113.9	759.65	91.9	36.0	94.9	29.4 ± 9.8
32-21	122.0	813.16	94.2	36.7	95.2	32.2 ± 10.5
32-25	120.8	805.15	92.9	36.2	94.7	30.5 ± 10.4
32-26	122.5	816.59	94.0	36.6	95.2	31.1 ± 10.6
32-27	122.5	816.69	93.6	36.4	95.0	30.2 ± 10.2

Table S3. Summary of categorical criteria for nine variant effect prediction tools.

Tools (dbtype)	Categorical Prediction
SIFT (sift)	D: Deleterious (sift<=0.05); T: tolerated (sift>0.05)
PolyPhen 2 HDIV (pp2_hdiv)	D: Probably damaging (>=0.957), P: possibly damaging (0.453<=pp2_hdiv<=0.956); B: benign (pp2_hdiv<=0.452)
PolyPhen 2 HVar (pp2_hvar)	D: Probably damaging (>=0.909), P: possibly damaging (0.447<=pp2_hdiv<=0.909); B: benign (pp2_hdiv<=0.446)
LRT (lrt)	D: Deleterious; N: Neutral; U: Unknown
MutationTaster (mt)	A" ("disease_causing_automatic"); "D" ("disease_causing"); "N" ("polymorphism"); "P" ("polymorphism_automatic"
MutationAssessor (ma)	H: high; M: medium; L: low; N: neutral. H/M means functional and L/N means non-functional
FATHMM (fathmm)	D: Deleterious; T: Tolerated
MetaSVM (metasvm)	D: Deleterious; T: Tolerated
MetaLR (metalr)	D: Deleterious; T: Tolerated

Table S4. The pLI relating scores for 13 analyzed genes

Analyzed Gene	Transcript	pLI_Score	pNull_Score	pRec_Score
NR5A1	ENST00000373588	9.90E-01	5.80E-07	1.04E-02
TTC8	ENST00000380656	1.18E-06	2.39E-03	9.98E-01
EDA	ENST00000374552	9.74E-01	2.75E-05	2.61E-02
UPK3A	ENST00000216211	1.08E-10	9.75E-01	2.53E-02
STK11	ENST00000326873	9.93E-01	1.90E-07	6.60E-03
APC	ENST00000457016	1.00E+00	4.65E-34	5.56E-11
GAMT	ENST00000447102	9.48E-03	1.62E-01	8.28E-01
CHM	ENST00000357749	9.99E-01	1.97E-09	1.01E-03
ATXN3	ENST00000393287	1.06E-01	5.08E-05	8.94E-01
BCKDHA	ENST00000269980	9.82E-09	2.42E-01	7.58E-01
IGF2	ENST00000434045	4.41E-02	9.65E-02	8.59E-01
INS-IGF2	ENST00000397270	5.58E-02	7.15E-02	8.73E-01
FBN3	ENST00000600128	5.44E-42	1.20E-04	1.00E+00

Table S5. Summary of diagnostic rates for several recent WES/WGS studies

Published Year	Journal	Strategy	Solved	Sequenced	Diagnostic Yield (%)	Reference
2013	NEJM	WES	62	250	24.8	Ref30
2014	AJHG	WES	146	264	55.3	Ref31
2015	Nat Genet.	WGS	33	156	21.2	Ref32
2017	JAMA Pediatr.	WES	23	44	52.3	Ref33
2017	JAMA Pediatr	WES	102	278	36.7	Ref34
		Critical trio WES	32	63	50.8	
2018	NPJ Genom Med	WGS	18	42	42.9	Ref35

Fam5-1

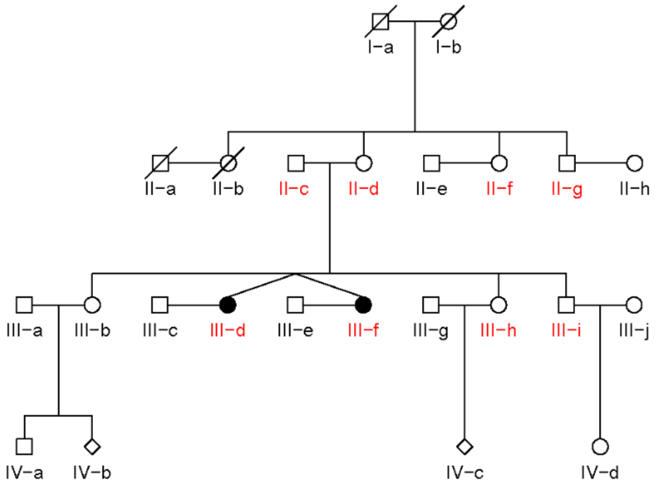

Fam7

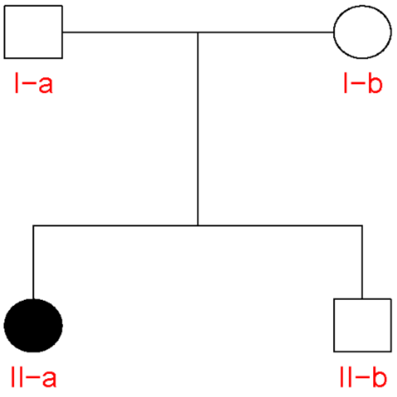

Fam8

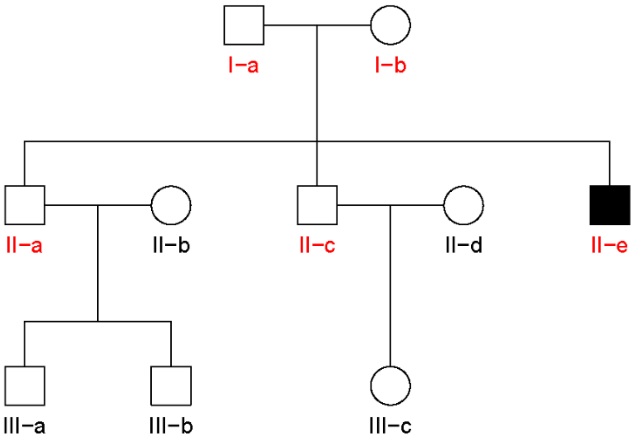

Fam10-1

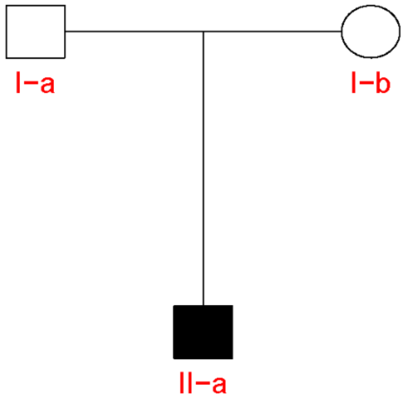

Fam10-2

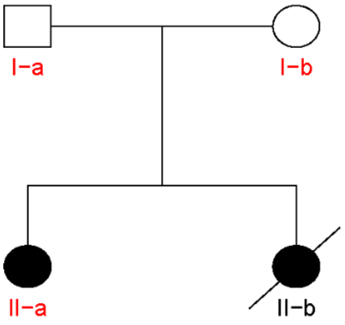

Fam13

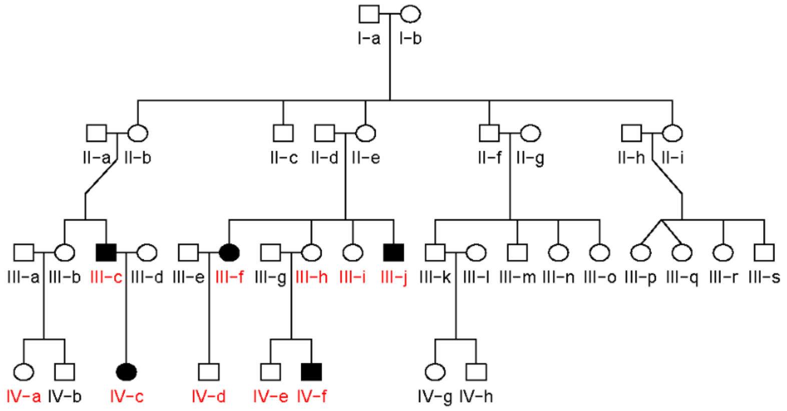

Fam20

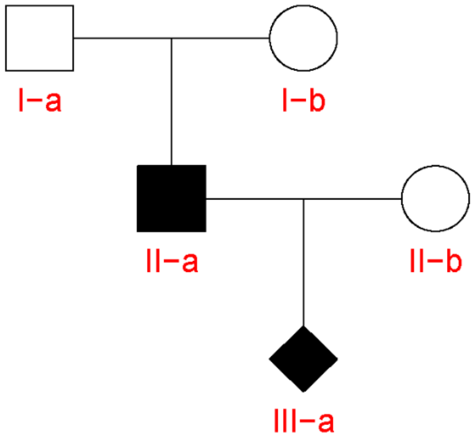

Fam21

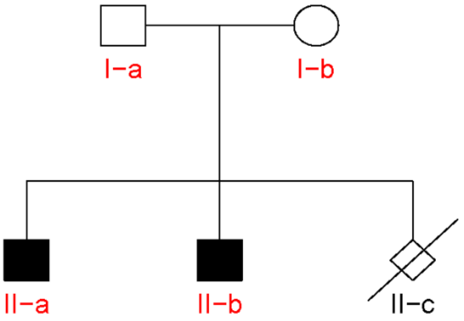

Fam22-1

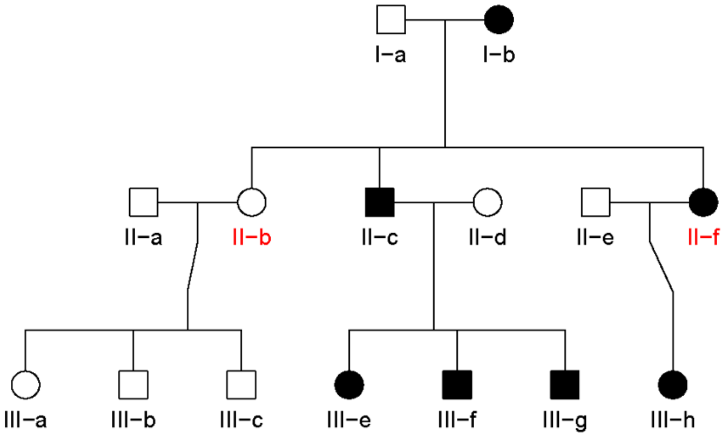

Fam22-2

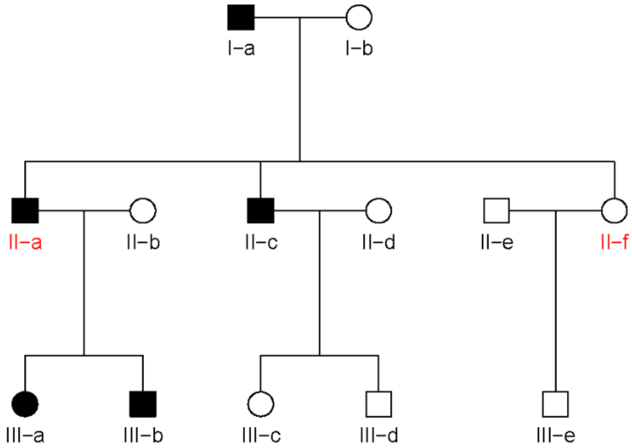

Fam24

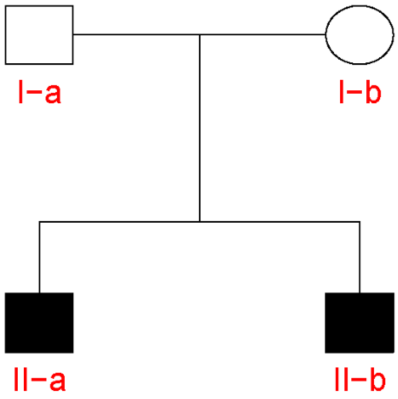

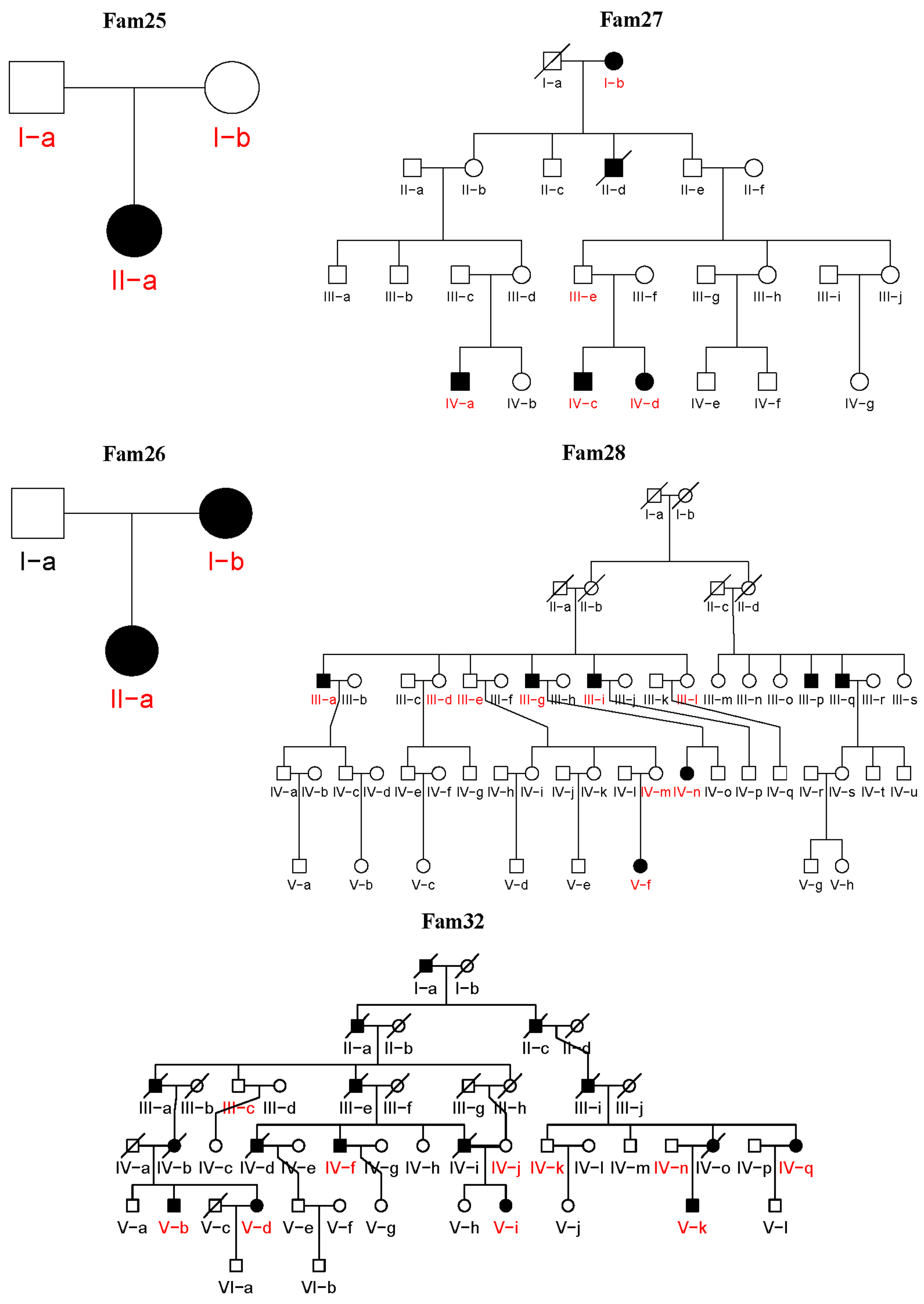

Fig. S1. Pedigrees of 16 families with rare disorders. Squares represent males and circles represent females. Squares or circles filled with black represent the affected. Squares or circles with a slash represent the deceased. The individuals with red text are selected for sequencing.

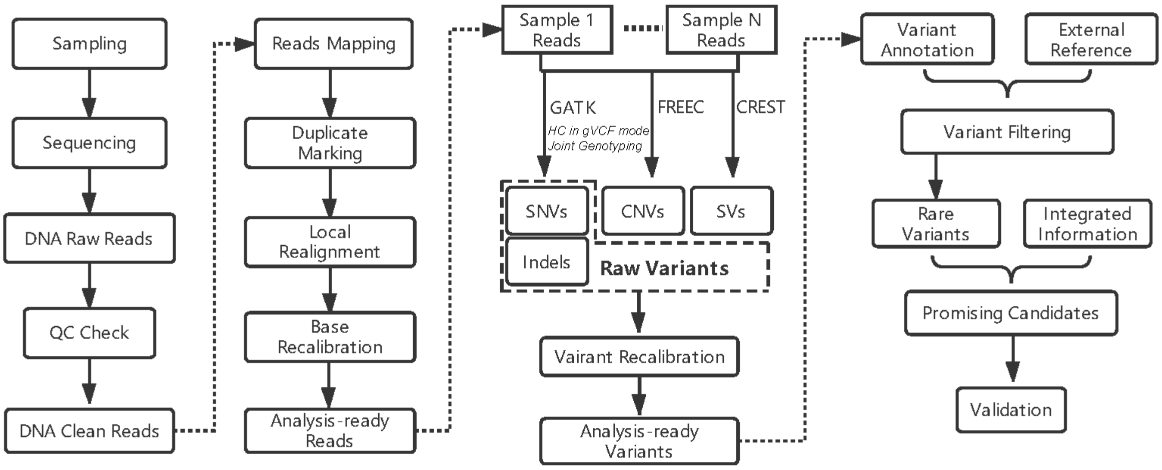

Fig. S2. Bioinformatics pipeline of read alignment and variant calling. Briefly, the Genome Analysis Toolkit (GATK) was adopted for read alignment and variant calling. Variant calling was performed for each sample individually using the HaplotypeCaller in gVCF mode. Then, joint variant calling was conducted on these gVCFs to increase genotyping accuracy. The variants produced from the joint-call cohort were used for subsequent variant filtration.

Fam5-1

(Gene: NR5A1(Chr9:127265357 C>T; Splicing); Inheritance Mode: Dominant)

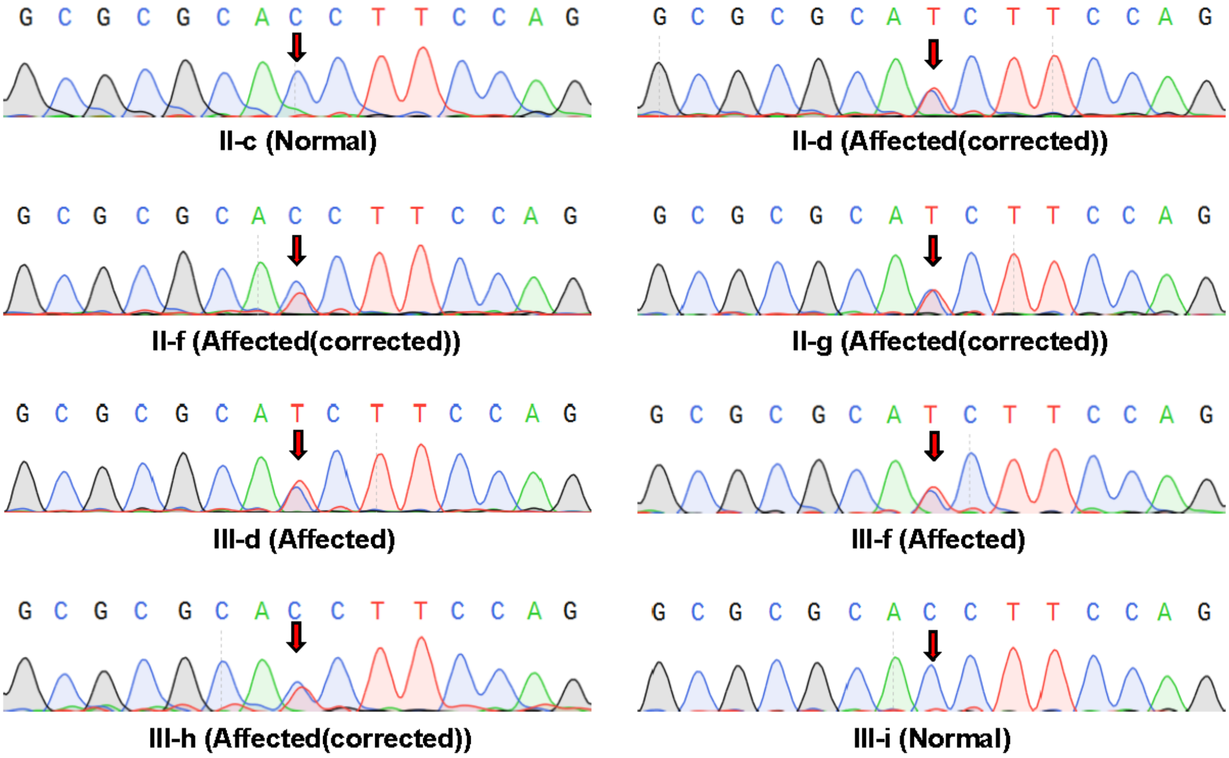

Fam7
(Gene: BCKDHA(Chr19:41928938 C>T; Missense); Inheritance Mode: *de novo*)

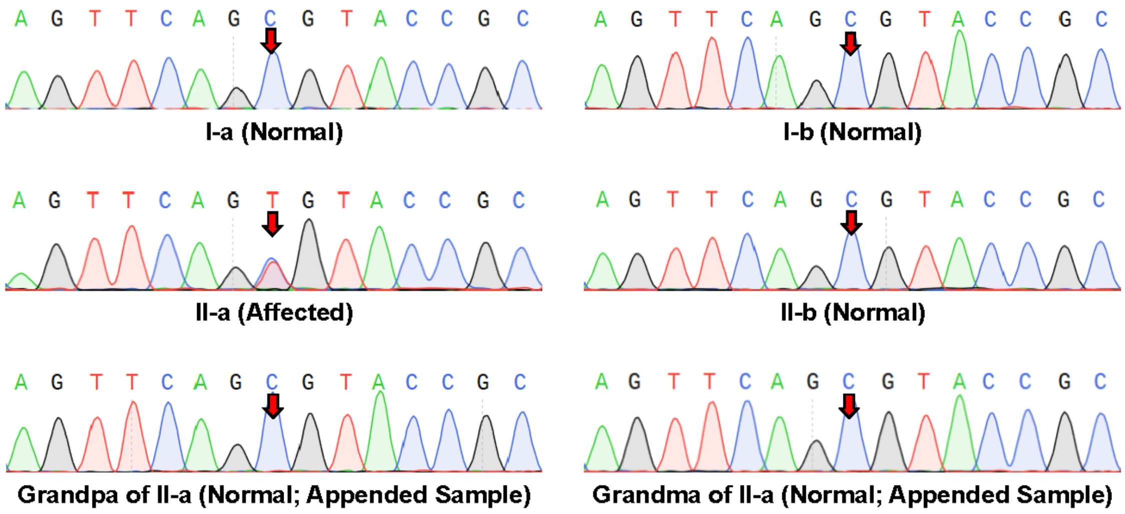

Fam7
(Gene: IGF2, INS-IGF2(Chr11:2170355 C>T; Splicing); Inheritance Mode: Imprinted (paternal expressed))

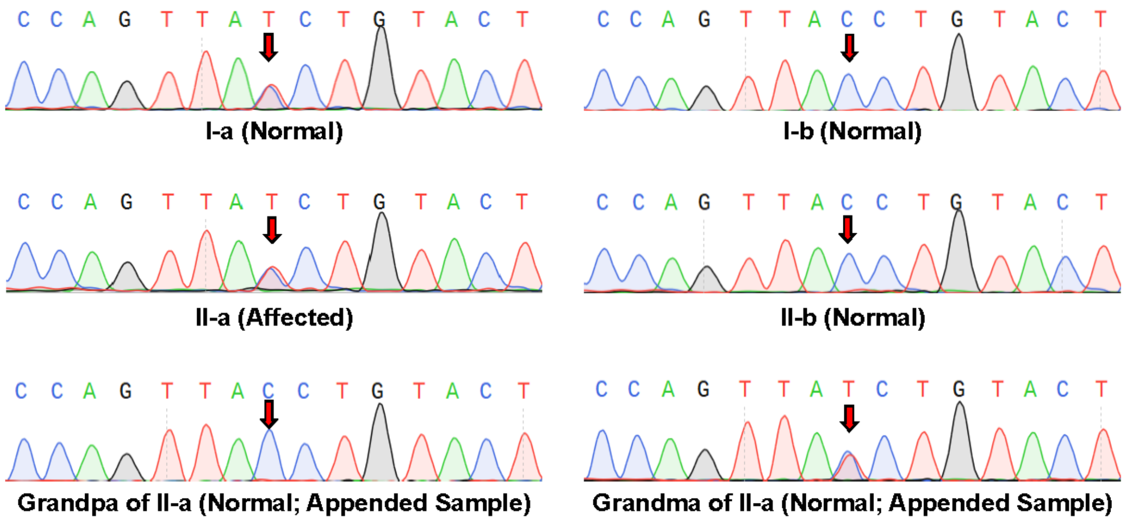

Fam10-1
(Gene: TTC8(Chr14:89327564 T>C; Splicing); Inheritance Mode: Recessive)

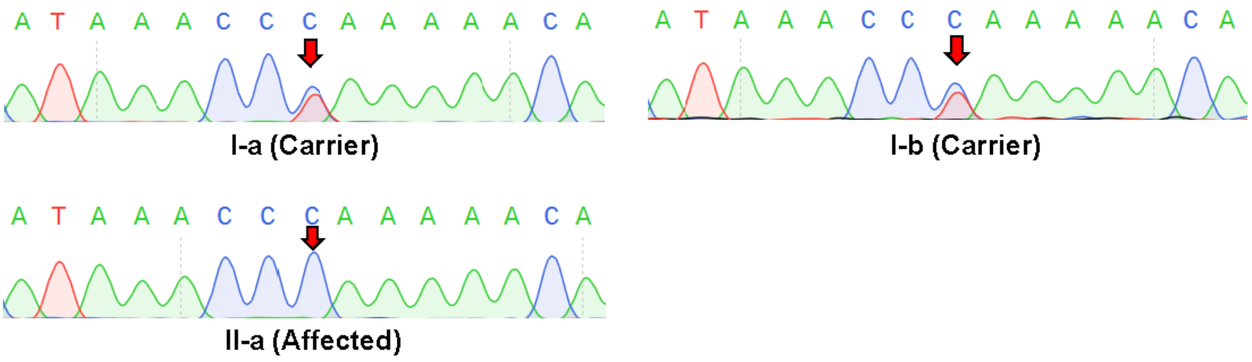

Fam13

(Gene: EDA(ChrX:69176954 A>C; Missense); Inheritance Mode: X-linked Recessive)

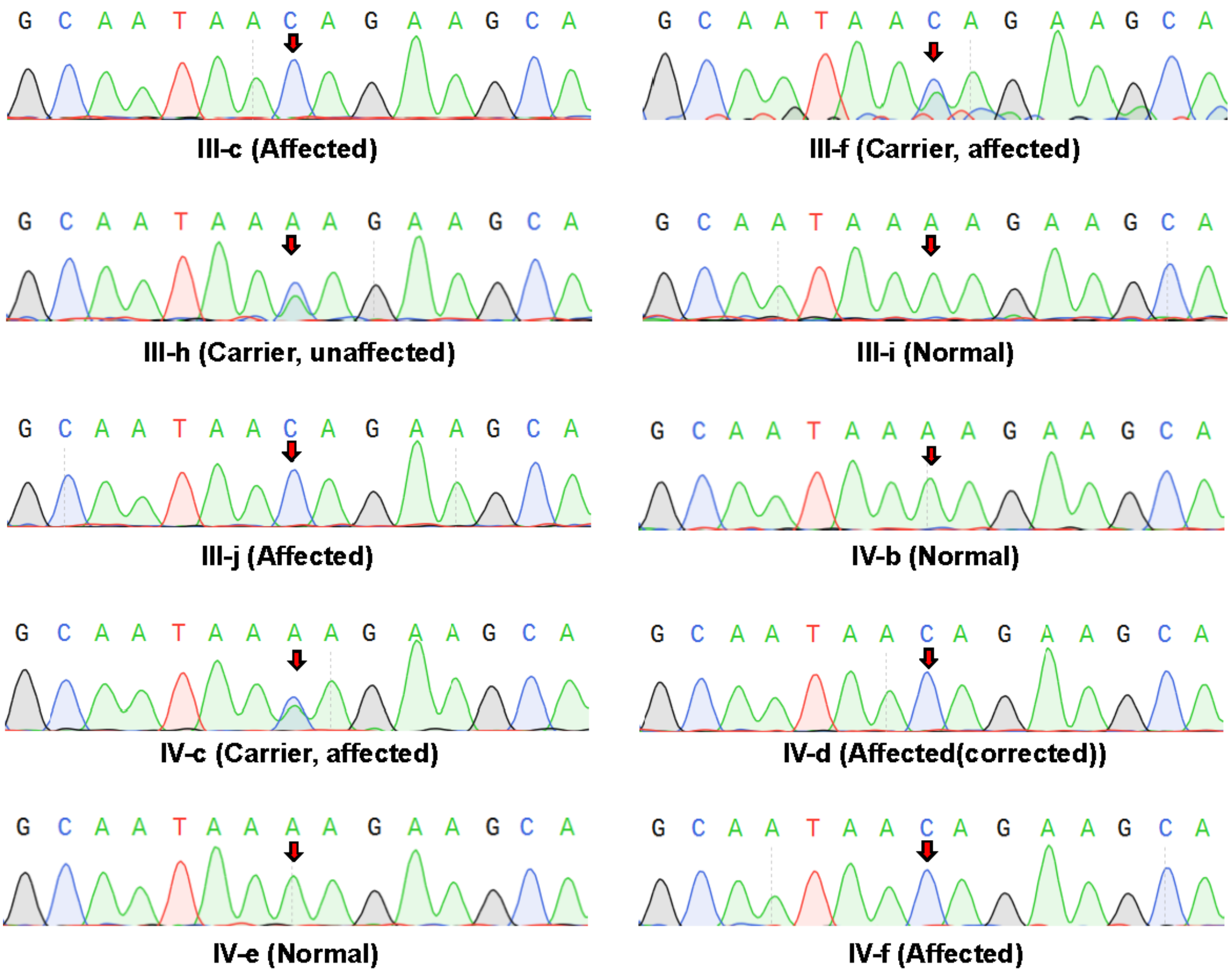

Fam21

(Gene: UPK3A(Chr22:45683310 delCT; Frameshift_deletion); Inheritance Mode: Compound Heterozygous)

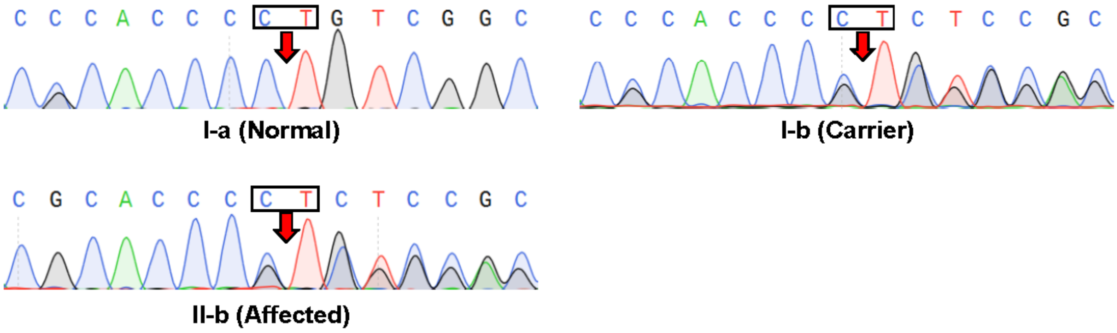

Fam21

(Gene: UPK3A(Chr22:45684998 G>A; Nonsense); Inheritance Mode: Compound Heterozygous)

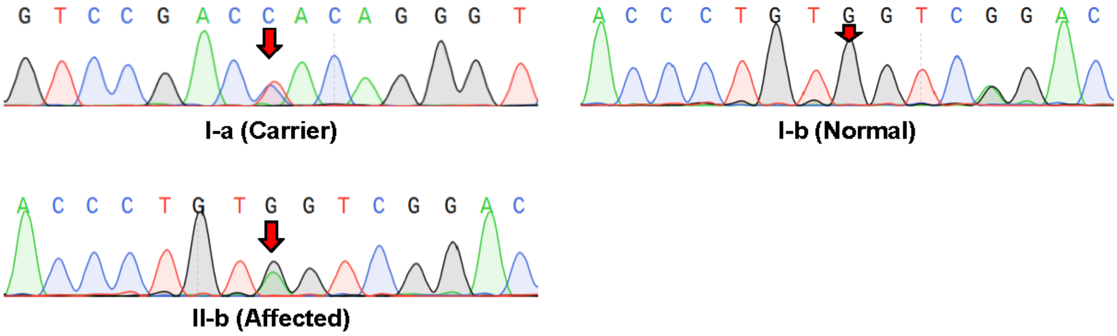

Fam22-1

(Gene: STK11(Chr19:1219406 C>A; Missense); Inheritance Mode: Dominant)

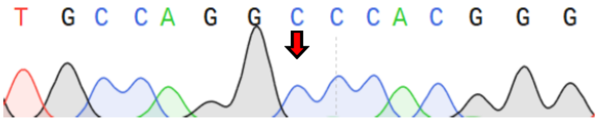

II-b (Normal)

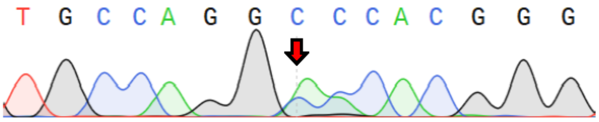

II-f (Affected)

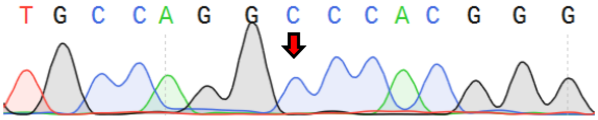

I-a (Normal; Appended Sample)

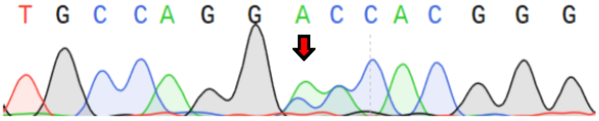

II-c (Affected; Appended Sample)

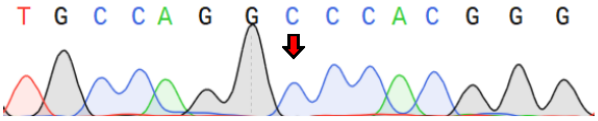

II-d (Normal; Appended Sample)

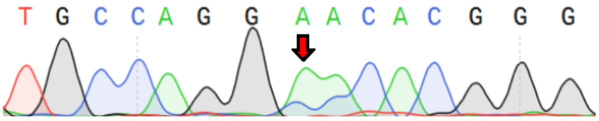

III-e (Affected; Appended Sample)

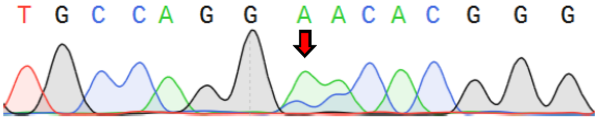

III-f (Affected; Appended Sample)

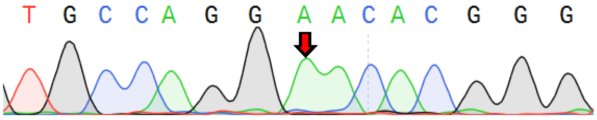

III-g (Affected; Appended Sample)

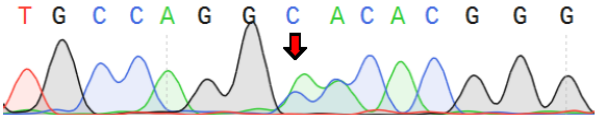

III-h (Affected; Appended Sample)

Fam22-2

(Gene: APC(Chr5:112128143 C>T; Nonsense); Inheritance Mode: Dominant)

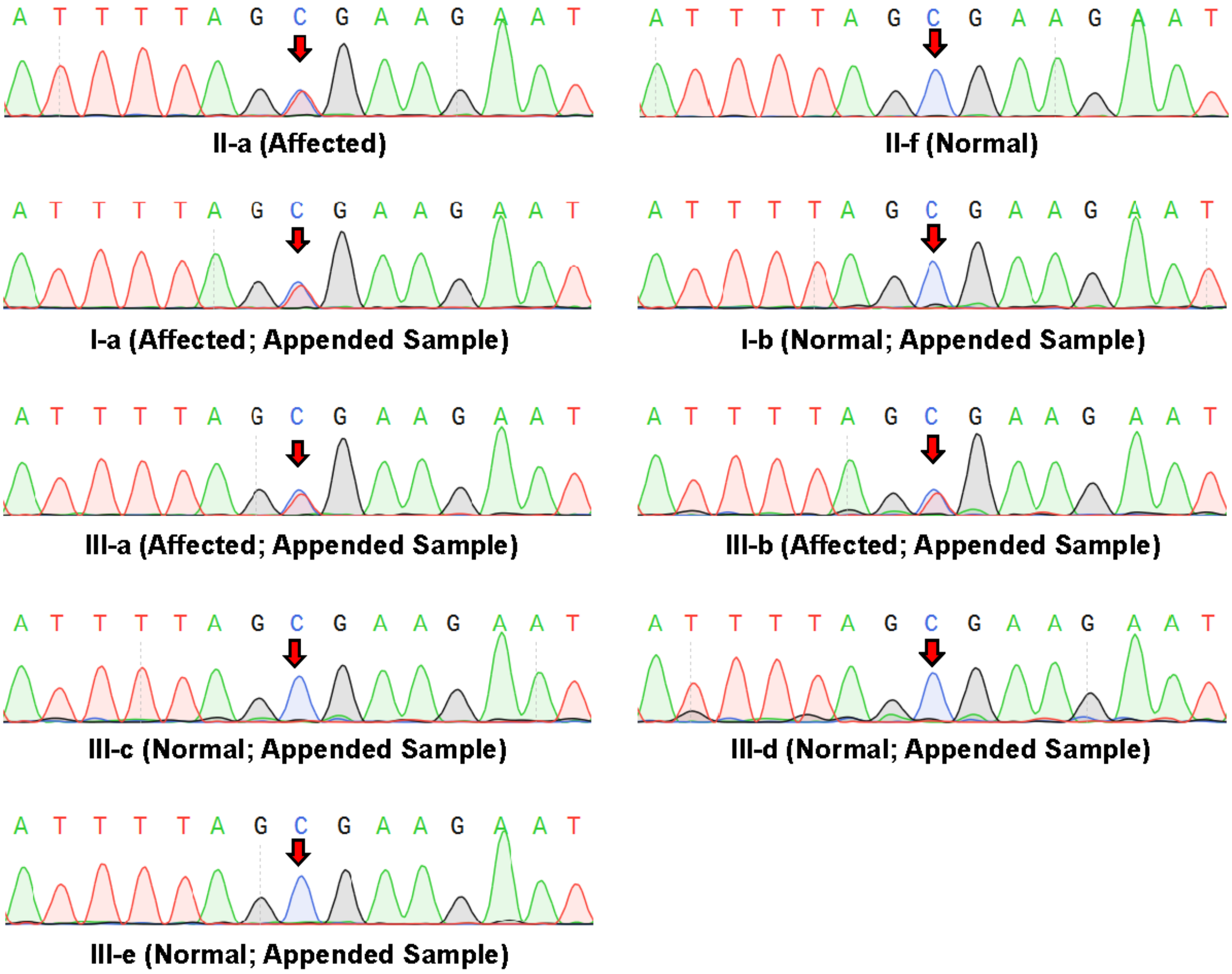

Fam24

(Gene: GAMT(Chr19:1399922 A>C; Missense); Inheritance Mode: Recessive)

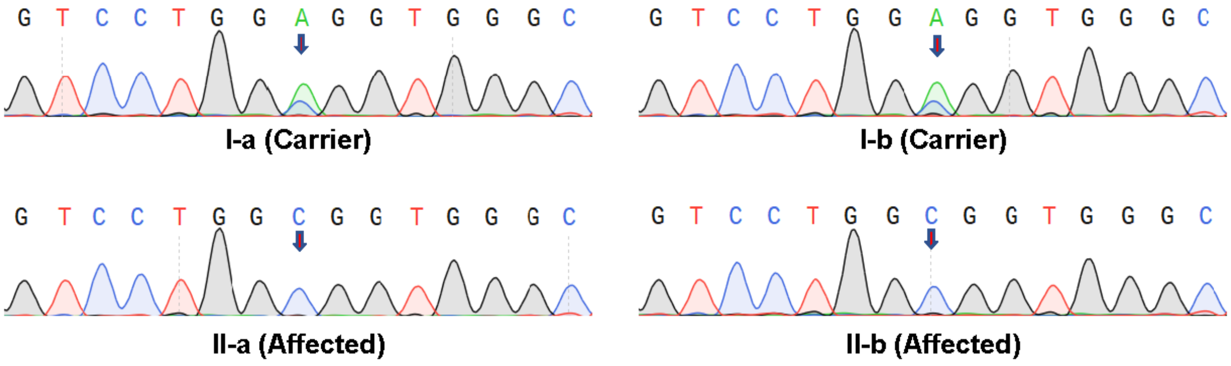

Fam25

(Gene: FBN3(Chr19:8188820 C>T; Missense); Inheritance Mode: Recessive)

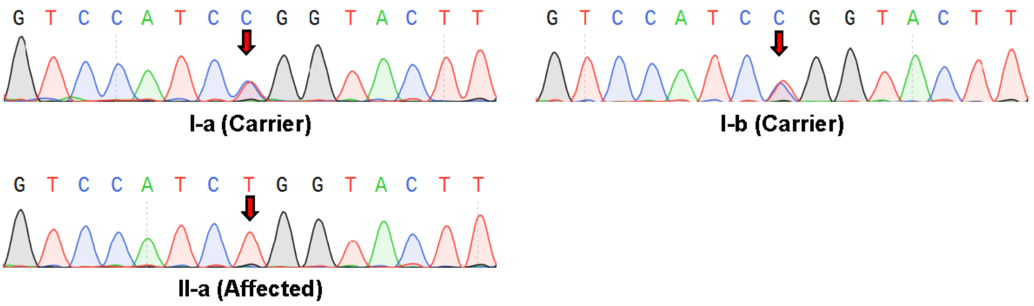

Fam28

(Gene: CHM(ChrX:85213886 G>A; Nonsense); Inheritance Mode: X-linked Recessive)

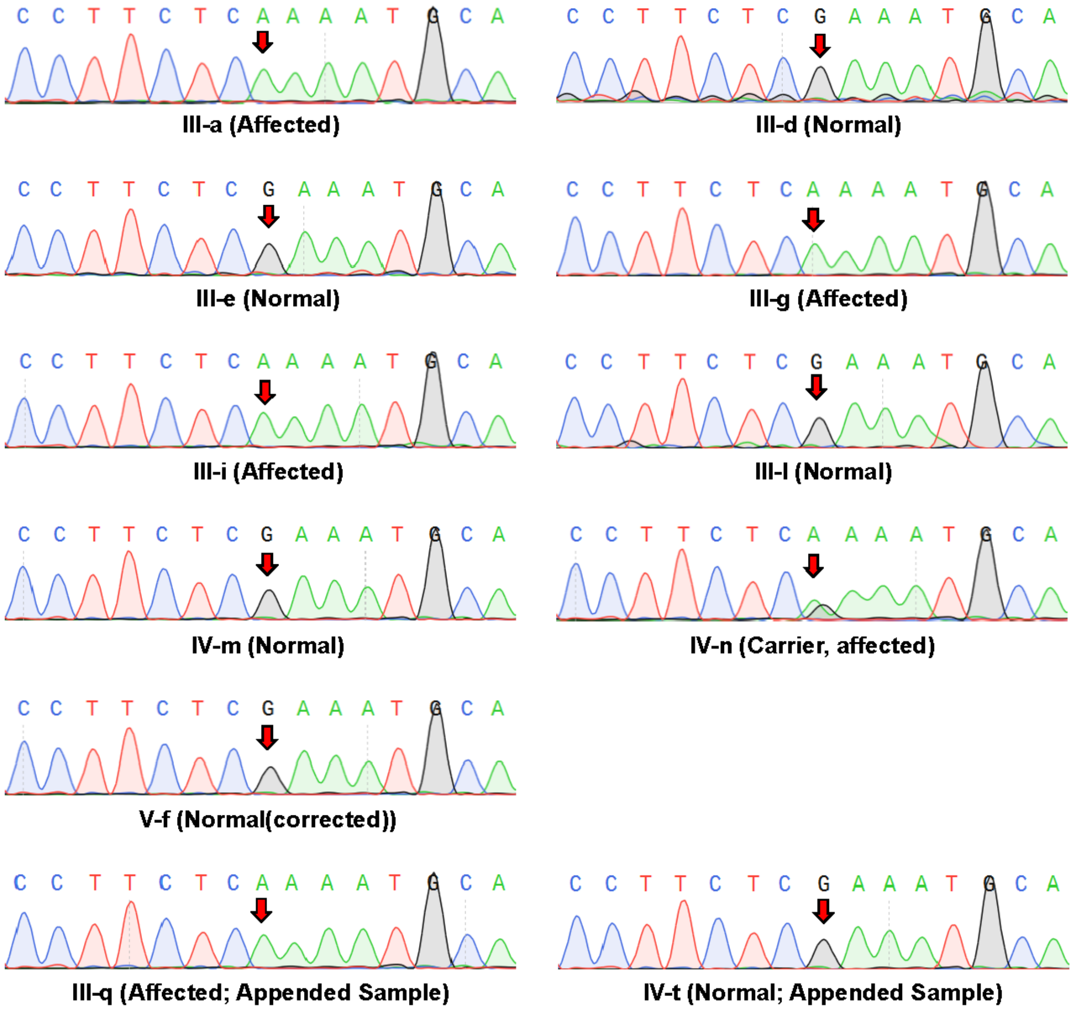

Fig. S3. PCR and Sanger validation results. For a specific Sanger sequencing result, each of four bases is recorded at top with its own representative color as shown in the figure. Below, the single peak indicates the homozygous state of the above base and bimodal peak indicates the heterozygous state of bases interpreted by the colors of peaks. Red arrow indicates the position of target variant in Sanger validation sequence.

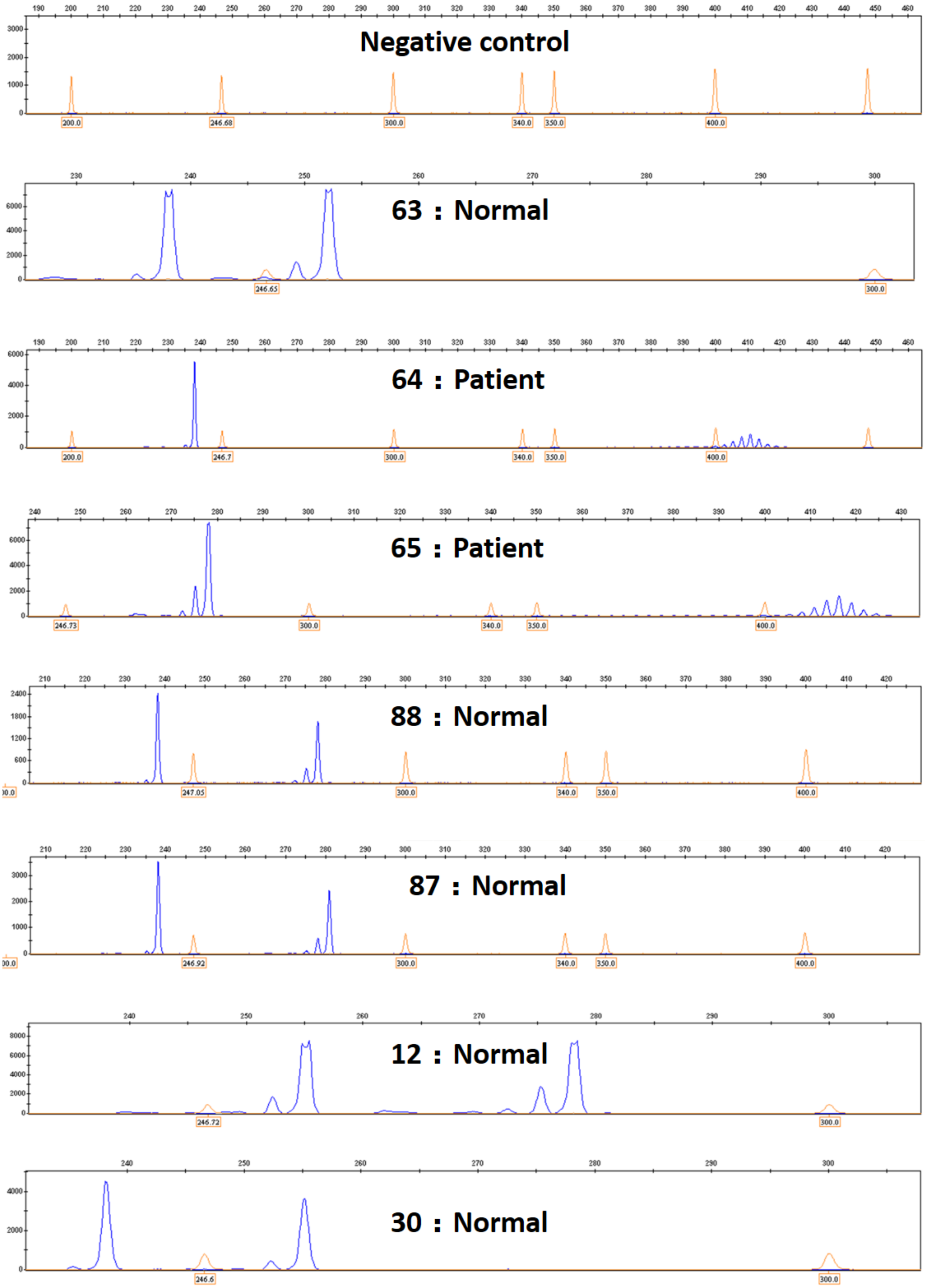
Negative control
63 : Normal
64 : Patient
65 : Patient
88 : Normal
87 : Normal
12 : Normal
30 : Normal

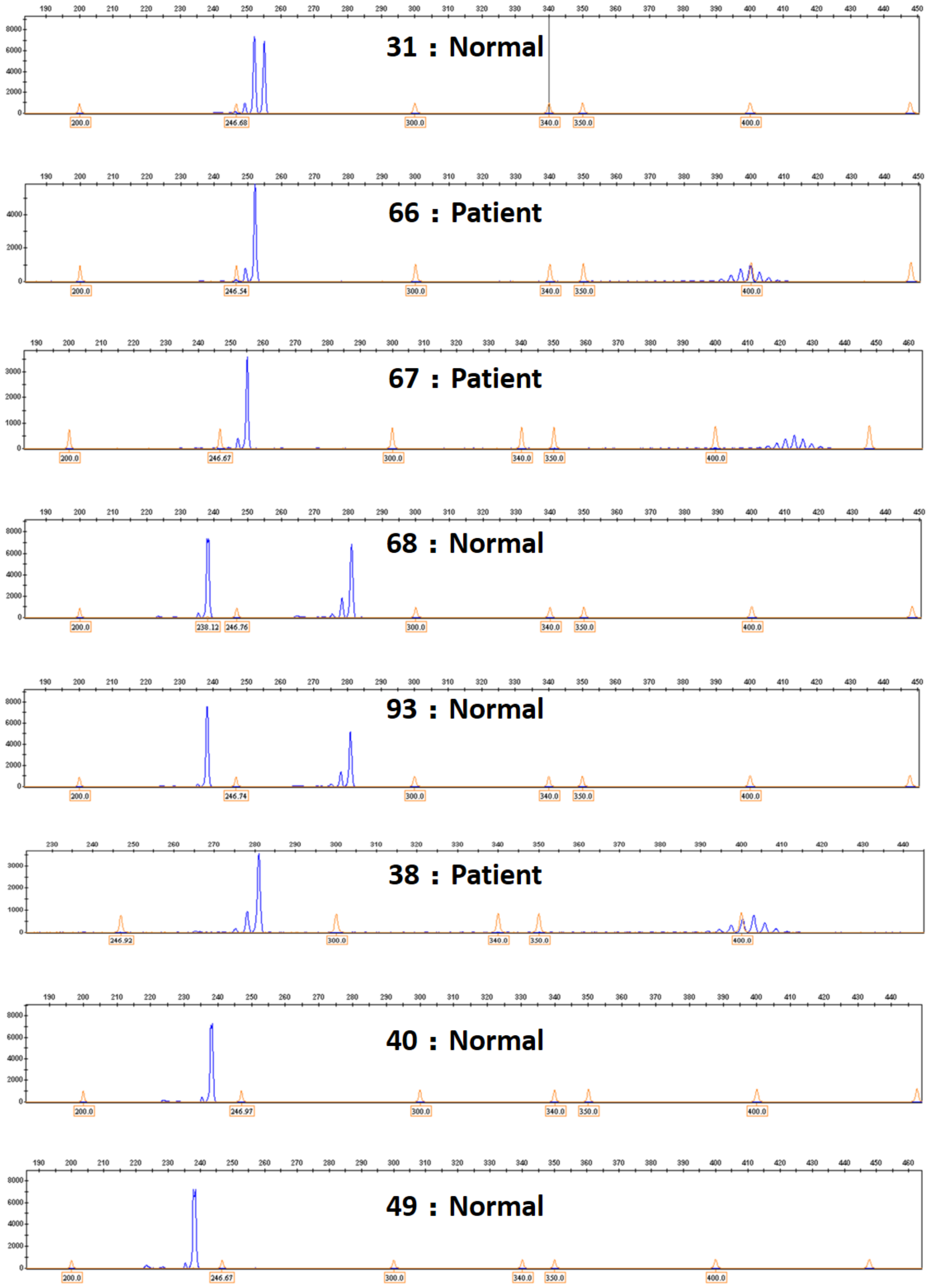
31 : Normal
66 : Patient
67 : Patient
68 : Normal
93 : Normal
38 : Patient
40 : Normal
49 : Normal

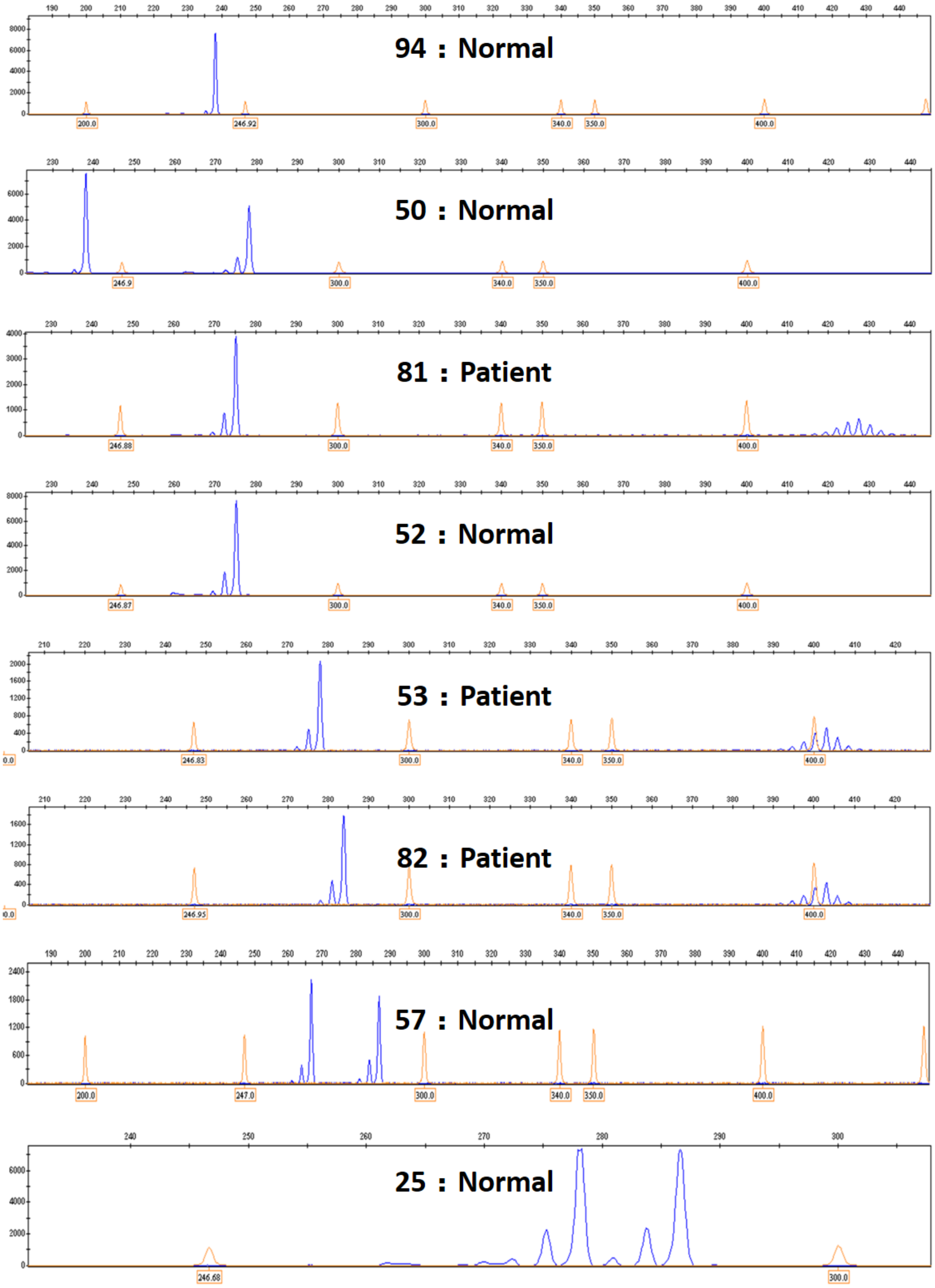

Fig. S4. HPCE analysis of SSR in ATXN3. The horizontal coordinate represents the length of PCR products, and the vertical coordinate represents the concentration of PCR products. The yellow peak is the DNA marker that marked corresponding location information, which assists to normalize the length of input PCR products. Blue peaks are our input PCR products from normal people and patients. Single peak represents homozygous genotype while bimodal peaks represent heterozygous genotype of people. The PCR products of normal people are within 26-40 CAG repeats, while patients have one longer unstable PCR products within more than 60 CAG repeats.
